# Awake prone positioning for patients with COVID-19-related respiratory failure: a systematic review and meta-analysis

**DOI:** 10.1007/s11739-023-03434-1

**Published:** 2023-10-05

**Authors:** Mara Graziani, Andrea Galeazzo Rigutini, Diletta Bartolini, Laura Traballi, Lorenzo Luzi, Rossana Regina, Francesco Bossi, Carla Caponi, Cecilia Becattini

**Affiliations:** Internal, Vascular and Emergency Medicine-Stroke Unit, University of Perugia, Perugia, Italy

**Keywords:** COVID-19, SARS-CoV-2, Acute respiratory distress syndrome, Respiratory failure, Hypoxemic respiratory failure, Awake prone positioning

## Abstract

The role of awake prone positioning (aPP) in patients with acute hypoxemic respiratory failure is debated. We performed a systematic review and meta-analysis to evaluate the role of aPP in acute respiratory failure related to COronaVIrus Disease-19 (COVID-19). Studies reporting on the clinical course of patients with acute respiratory failure related to COVID-19 treated or not treated by aPP were included in the systematic review and meta-analysis (ProsperoID: CRD42022333211). The primary study outcome was the composite of in-hospital death or orotracheal intubation; the individual components of the primary outcome were secondary study outcomes. The composite of in-hospital death or orotracheal intubation was available for 6 studies (1884 patients), five randomized and one prospective; a significant reduction in the risk of this outcome was observed in patients treated vs. not treated by aPP (33.5% vs. 39.8%; OR 0.73, 95% CI 0.60–0.89; *I*^2^ 0%). In-hospital death was reported in 34 studies (6808 patients) and occurred in 17.4% vs. 23.5% of patients treated or not treated with aPP (random effect OR 0.60, 95% CI 0.46–0.79; *I*^2^ 59%); orotracheal intubation was observed in 25.8% vs. 32.7% of patients treated or not treated with aPP (27 studies, 5369 patients; random effect OR 0.85, 95% CI 0.56–1.27; *I*^2^ 84%). aPP reduces the risk for death or orotracheal intubation in patients with acute respiratory failure related to COVID-19. Further studies should be conducted to confirm the clinical benefit of aPP outside the ICU.

*Registration* Prospero ID: CRD42022333211.

**Supplementary Information:**

The online version contains supplementary material available at 10.1007/s11739-023-03434-1.

## Introduction

Coronavirus 19 infection (COVID-19) is responsible for severe acute respiratory syndrome (SARS-CoV-2) that can evolve to progressive hypoxemic respiratory failure and to severe acute respiratory distress syndrome (ARDS) in about 17% of unvaccinated patients [1].

In the pre-COVID-19 era, a number of studies demonstrated a significant improvement in oxygenation and pulmonary mechanics by the use of prone positioning in patients requiring invasive mechanical ventilation for ARDS [2]. Prone positioning allows more even distribution of the gas–tissue ratios along the dependent–non-dependent axis and a more homogeneous distribution of lung stress and strain. In a randomized study, early and prolonged prone positioning sessions (of at least 16 h) were associated with a reduction of about 61% in mortality with no increase in adverse events in intubated patients admitted to intensive care units (ICUs) [3]. These results were later confirmed in several meta-analyses [4]. Based on this evidence, prone positioning has been used for more than 40 years to improve oxygenation in intubated patients with ARDS [5] and is now strongly advocated in the management of moderate-to-severe ARDS [6].

During SARS-CoV-2 pandemic, several cohort studies claimed an effect of prone positioning in reducing mortality in intubated patients with SARS-CoV-2-related ARDS [7]. The World Health Organization guidelines recommend prone positioning for the management of intubated COVID-19 patients with ARDS as a beneficial practice despite the lack of evidence from randomized clinical trials [8]. The use of prone positioning has also been proposed in awake COVID-19 patients with severe respiratory failure, to improve oxygenation and reduce progression to orotracheal intubation [9]. However, conflicting results are currently available on the role of awake prone positioning in this setting. As for today, the effectiveness of prone positioning in reducing progression to intubation and mortality in awake COVID-19 patients remains unclear [10, 11].

The aim of this systematic review and meta-analysis is to assess the role of awake prone positioning in reducing death or orotracheal intubation in patients with acute respiratory failure related to COVID-19.

## Methods

A protocol for this study was prospectively developed detailing the specific objectives, criteria for study selection, approach to assess study quality, outcomes, and statistical methods (PROSPERO registration number CRD42022333211).

This study was conducted according to the methodology suggested by the Providing Innovative Service Models and Assessment (PRISMA) criteria [12].

### Data sources and searches

We performed an unrestricted search in Pubmed, Web of Science, OVID, MedRxiv, and ClinicalTrials.gov, from inception through March 22, 2023. No language restrictions were applied. Reference lists of retrieved articles and review articles were manually searched for other relevant studies. The search strategy is reported in the Supplementary material.

### Study selection

Seven reviewers (M.G., A.G.R., D.B., F.B., L.L., R.R., C.C.) performed study selection independently, with disagreements solved through discussion and the opinion of an additional reviewer (C.B.). Studies in patients with COVID-19 were considered eligible for the systematic review and meta-analysis if they met the following predetermined criteria: (a) inclusion of hospitalized and non-intubated patients with acute respiratory failure related to COVID-19; (b) inclusion of patients treated and non-treated with prone positioning; and (c) reporting on study outcome events in patients treated and not treated with prone positioning. Studies were excluded in case of (a) diagnosis of COVID-19 not confirmed by molecular swab testing; (b) case reports; and (c) inclusion of less than 20 patients.

### Study outcome

The primary study outcome was the composite of in-hospital death or need for endotracheal intubation.

Secondary study outcomes were the individual components of the primary outcome: in-hospital death and need for orotracheal intubation.

Need for orotracheal intubation was reported according to the definition used in the individual studies.

For duplicate publications, the most complete was considered.

### Data extraction and risk of bias assessment

For each study, the following data were extracted independently by two authors: general study data (design, year of publication), population characteristics (mean age, gender, severity of respiratory failure, use of non-invasive ventilation), clinical setting (intensive care unit [ICU], non-ICU), data on prone positioning (number of patients and duration), and study outcomes (in-hospital death or endotracheal intubation and the individual components of the composite outcome in patients treated and not treated with prone positioning).

Risk of bias of selected studies was independently assessed by three reviewers (A.G.R., M.G., and D.B.) using the Cochrane Collaboration’s tools (ROBIS-I for non-randomized and ROB-II for randomized studies [RCTs]), which cover the following bias domains: selection bias, performance bias, detection bias, attrition bias, and reporting bias [13]. Each potential source of bias was graded as high, low, or some concerns, which determined whether the studies were considered at high, low, or moderate risk of bias.

We resolved disagreements in study data extraction and risk of bias assessment by consensus or by discussion with an additional reviewer (C.B.).

### Statistical analysis

Study outcomes were compared in patients treated or not treated by awake prone positioning.

Estimates of pooled effect sizes were obtained by the Mantel–Haenszel method. Pooled odds ratios (ORs) were reported with 95% confidence intervals (CIs). Forest plots were created for each outcome. Statistically significant heterogeneity was considered present at *I*^2^ > 50% or Cochran’s chi^2^ test *p* < 0 [14, 15]. Results were reported according to fixed-effects model in the absence of significant heterogeneity and to random-effects model in the presence of significant heterogeneity. Correction for zero cells was performed by adding 0.5 to all zeros, when risk measures were not estimable [16]. Publication bias was assessed visually by funnel plots inspection.

To assess potential sources of heterogeneity, pre-defined sensitivity analyses were conducted as it follows: (i) studies including patients admitted to ICU vs. non-ICU; (ii) duration of prone positioning lower than 6 hours, between 6 and 12 h or ≥ 12 h/day; (iii) studies recruiting from 1/2020 to 12/2020 vs. beyond January 2021; (iv) retrospective vs. prospective vs. RCTs; (v) follow-up lower than seven days vs. between 7 and 14 days vs. between 14 and 28 days vs. over 28 days; (vi) studies including ≤ 100 patients vs. > 100 patients; (vii) studies with low risk of bias vs. moderate vs. high; and (viii) studies excluding vs. including do-not-resuscitate (DNR) patients.

The statistical analyses, forest plots, and funnel plots were produced by using Review Manager release 5.4 (The Cochrane Collaboration, Oxford, England) and STATA/SE 12 (StataCorp LP, College Station, TX, USA).

## Results

Overall, 9443 studies were retrieved by electronic searches and 111 were selected as candidates for inclusion in the study after title and abstract review. After full text review, 34 studies (6808 patients) were finally identified for inclusion in the systematic review and meta-analysis [17–50]. The flow of study selection is reported in e-Fig. 1 reported on Supplementary material.

The main features of included studies are reported in e-Table 1. Six studies were RCTs and 10 were prospective and 18 retrospective cohorts. Fifteen studies reported on non-intubated patients in the setting of ICU [17, 19–22, 25, 27, 30, 33, 36, 46, 48, 49] and two studies a mixed ICU–non-ICU population [24, 40]. The number of included patients varied across the included studies between 20 and 1121. The duration of follow-up was available for 31 studies and was less than 7 days, between 7 and 14 days, between 14 and 28 days, and more than 28 days, in six, ten, six, and six studies, respectively.

The proportion of patients treated with awake prone positioning, as well as the methods for prone positioning (duration, number of sessions), largely varied across the included studies (e-table 1).

The risk of bias for the included RCTs was low and moderate in four and two studies each (e-table 4); the risk of bias was moderate for the majority of cohort studies (e-table 4). Overall, the risk of bias was critical in two and serious in four studies.

### Clinical course with vs. without awake prone positioning

**Fig. 1:**
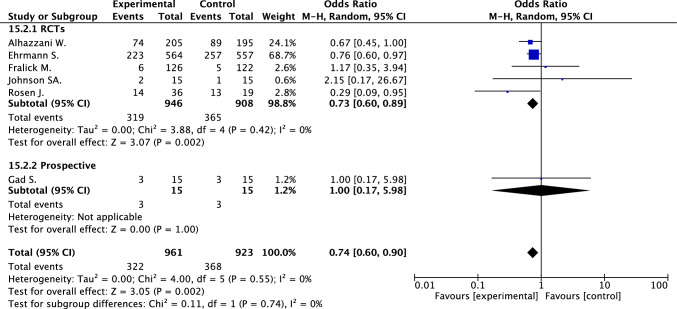
In-hospital death or orotracheal intubation in patients treated and not treated by awake prone positioning

The composite of in-hospital death or orotracheal intubation was reported in two studies and was available after contact with the authors in additional four studies [17, 24, 25, 33, 34, 43]. Five of these studies were randomized, open-label studies and one prospective study, overall including 1884 patients, 961 treated with and 923 without awake prone positioning. The analysis of these studies showed a significant reduction in the incidence of in-hospital death or orotracheal intubation in favor of awake prone positioning (fixed effect OR 0.74, 95% CI 0.60–0.90; *I*^2^ 0%) (Fig. 1).

Incidences of in-hospital death and of orotracheal intubation in each study by use of awake prone positioning are reported in Table 1.

**Table 1:** In-hospital death and orotracheal intubation in the individual studies by intervention group (aPP vs. not aPP)

Author, year	Death in patients treated by aPP *n*/*N* (%)	Death in patients not treated by aPP *n*/*N* (%)	Orotracheal intubation in aPP patients *n*/*N* (%)	Orotracheal intubation in non-aPP patients *n*/*N* (%)	RCT	DNR patients (%)
Alhazzani W., 2022 [17]	46/205 (22.4)	46/195 (23.5)	70/205 (34.1)	79/195 (40.5)	Yes	Excluded
Altinay M., 2021 [18]	9/25 (36.0)	16/23 (69.8)	8/25 (32.0)	19/23 (82.6)	No	n.a
Ates I., 2021 [19]	0/97 (0.0)	4/47 (4.12)	7/97 (7.2)	12/47 (25.5)	No	n.a
Bahloul M., 2021 [20]	14/21 (66.7)	12/17 (70.6)	9/21 (42.8)	4/17 (23.5)	No	n.a
Barker J., 2021 [21]	1/10 (10.0)	4/10 (40.0)	6/10 (60.0)	5/10 (50.0)	No	n.a
Burton-Papp HC. 2020 [22]	0/20 (0.0)	0/20 (0.0)	7/20 (35.0)	0/20 (0.0)	No	n.a
Coppo A., 2020 [23]	0/47 (0.0)	0/9 (0.0)	13/47 (27.6)	0/9 (0.0)	No	n.a
Ehrmann S., 2021 [24]	117/564 (20.7)	132/557 (23.7)	185/564 (32.6)	223/557(40.0)	Yes	Included (8)
Ferrando C., 2020 [25]	8/49 (16.3)	17/122 (13.9)	22/55 (40.0)	60/144 (41.7)	No	n.a
Fralick M., 2022 [26]	1/126 (0.80)	1/122 (0.81)	5/126 (3.9)	6/122 (4.9)	Yes	Included
Gad S., 2021 [27]	3/15 (20.0)	3/15 (20.0)	3/15 (20.0)	3/15 (20.0)	No	n.a
Graziani M, 2023 [28]	23/114 (20.1)	102/422(24.1)	39/114 (34.2)	32/422 (7.5)	No	Included (21)
Hallifax RJ, 2020 [29]	12/30 (40.0)	14/18 (77.7)	–	–	No	n.a
Hashemian SM., 2021 [30]	9/45 (20.0)	10/30 (33.3)	10/45 (22.2)	12/30 (40.0)	No	n.a
Hussain HT., 2021 [31]	1/25 (4.0)	2/25 (8.0)	–	–	No	n.a
Imran M., 2021 [32]	2/50 (4.0)	3/50 (6.0)	–	–	No	n.a
Jagan N., 2020 [33]	0/40 (0.0)	16/65 (40.0)	11/40 (27.5)	26/65 (40.0)	No	n.a
Jayakumar D., 2021 [34]	3/30 (10.0)	2/30 (6.7)	4/30 (13.3)	4/30 (13.3)	Yes	n.a
Johnson SA., 2021 [35]	2/15 (13.3)	0/15 (0.00)	2/15 (13.3)	1/15 (6.7)	Yes	n.a
Jouffroy R., 2021 [36]	4/40 (10.0)	94/339 (27.7)	4/40 (10.0)	200/339(58.9)	No	n.a
Liu X., 2020 [37]	0/13 (0.0)	0/16 (0.0)	*n*/*N* (%)	–	No	n.a
Musso G., 2022 [38]	10/81 (12.3)	59/162 (36.4)	8/81 (9.8)	44/162 (27.1)	No	Excluded
Padrao EMH., 2020 [39]	6/57 (10.5)	22/109 (20.2)	33/57 (57.9)	53/109 (48.6)	No	Excluded
Perez-Nieto OR. 2021 [40]	100/505 (1.9)	120/322 (36.3)	109/505 (21.5)	130/322 (40.4)	No	n.a
Proud’homme E. 2021 [41]	4/48 (8.3)	6/120 (5.0)	7/48 (14.5)	8/120 (6.6)	No	n.a
Qian ET., 2022 [42]	59/239 (24.7)	47/222 (21.1)	–	–	No	n.a
Rosen J., 2021 [43]	6/36 (16.7)	3/39 (7.7)	12/36(33.3)	13/39 (33.3)	Yes	Excluded
Simioli F., 2021 [44]	0/18 (0.0)	3/11 (27.3)	1/18 (5.5)	2/11 (18.1)	No	n.a
Syrma PB., 2021 [45]	2/30 (6.67)	4/15 (26.7)	–	–	No	n.a
Stilma W., 2021 [46]	91/438 (20.8)	62/296 (21.0)	–	–	No	n.a
Thompson A. 2020 [47]	3/25 (12.0)	0/40 (0.0)	13/25 (52.0)	4/40 (10.0)	No	Included
Tonelli R., 2021 [48]	5/38 (13.1)	17/76 (22.4)	7/38 (18.4)	30/76 (39.4)	No	Excluded
Vianello A., 2021 [49]	2/50 (0.0)	7/43 (16.8)	4/50(8.0)	12/43 (27.9)	No	Excluded
Zang X., 2020 [50]	10/23 (43.5)	28/37 (75.6)	8/23 (34.7)	4/37 (10.8)	No	n.a

*aPP* awake prone positioning, *DNR* do-not resuscitate, *RCT* randomized controlled trial

**Fig. 2:**
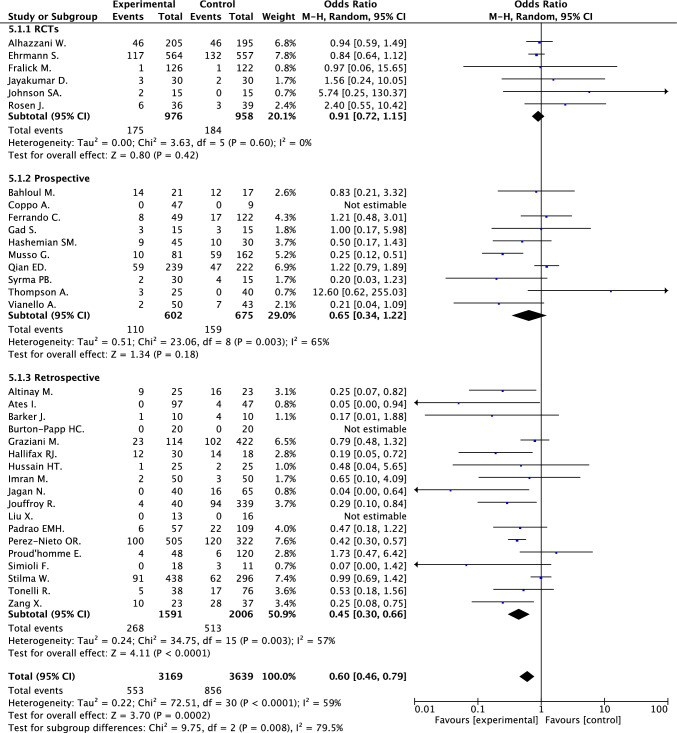
In-hospital death in patients treated and not treated by awake prone positioning according with the study design

In-hospital death was reported in 34 studies and occurred in 17.4% of 3169 patients receiving and in 23.5% of 3639 patients not receiving awake prone positioning, respectively; in these studies, awake prone positioning was associated with a significant reduction in mortality (random effect OR 0.60, 95% CI 0.46–0.79, *I*^2^ 59%) [17–50] (Fig. 2). These results were confirmed after correction for zero cells (random effect OR 0.61, 95% CI 0.49–0.76, *I*^2^ 50%) (e-Fig. 2).

**Fig. 3:**
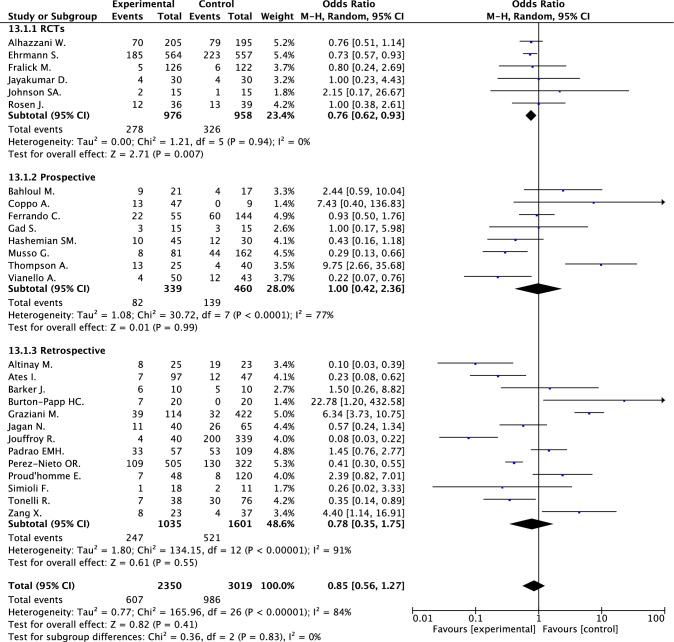
Orotracheal intubation in patients treated and not treated by awake prone positioning

Orotracheal intubation was reported in 27 studies (5369 patients) and occurred in 25.8% and in 32.7% of patients treated or not treated by awake prone positioning (random effect OR 0.85, 95% CI 0.56–1.27; *I*^2^ 84%) [17–28, 30, 33–36, 38–41, 43, 44, 47–50] (Fig. 3). These results were confirmed after correction for zero cells (random effect OR 0.78, 95% CI 0.53–1.15, *I*^2^ 83.3%) (e-Fig. 3).

No evidence of publication bias was observed at funnel plot inspection for these analyses (Supplementary material e-Fig. 4).

### Sensitivity analyses

Sensitivity analyses were conducted for the secondary outcomes (Table 2; e-Fig. 5 to e-Fig. 16).

**Table 2:** Sensitivity analyses for in-hospital death and orotracheal intubation

Sensitivity analyses	Number of studies	Death in patients treated aPP *n*/*N*	Death in patients not treated aPP *n*/*N*	OR	95% CI	*I*^2^ (%)
In-hospital death						
Study design						
RCTs	6	175/976	184/958	0.91	0.72–1.15	0
Prospective	10	110/602	159/675	0.65	0.34–1.22	65
Retrospective	18	268/1591	513/2006	0.45	0.30–0.66	57
Setting						
ICU	15	195/1071	309/1326	0.62	0.43–0.89	36
Non-ICU	17	141/1029	295/1434	0.62	0.36–1.36	65
ICU and non-ICU	2	217/1069	352/876	0.59	0.30–1.19	91
Duration of aPP						
< 6 h	18	225/1367	353/1630	0.63	0.44–0.90	33
6–12 h	8	141/903	308/1085	0.42	0.28–0.65	44
> 12h	3	23/112	36/184	0.85	0.25–2.86	68
Number of patients						
> 100	15	474/2641	745/3176	0.67	0.49–0.91	69
≤ 100	19	79/528	111/463	0.50	0.30–0.85	35
Duration of follow-up						
< 7 days	7	160/809	116/612	1.08	0.68–1.70	34
7–14 days	9	118/769	160/664	0.53	0.27–1.03	44
14–28 days	9	156/1064	452/1528	0.26	0.11–0.64	86
> 28 days	6	88/438	190/746	0.64	0.36–1.14	54
Period of study recruitment						
Before Dec-31-2020	25	390/2123	669/2687	0.57	0.41–0.79	59
After Dec-31-2020	9	73/633	127/681	0.59	0.31–1.13	52
Risk of bias						
High	9	262/1432	390/1748	0.82	0.57–1.18	62
Moderate	19	263/1553	421/1661	0.50	0.33–0.75	56
Low	6	28/184	45/230	0.48	0.22–1.08	37
DNR						
Included	3	143/703	234/1019	0.87	0.58–1.20	37
Excluded	6	75/467	154/624	0.55	0.29–1.06	66
Not known	28	364/2077	525/2337	0.58	0.43–0.80	49
Orotracheal intubation						
Study design						
RCTs	6	278/976	326/958	0.76	0.62–0.93	0
Prospective	8	82/339	139/460	1.00	0.42–2.36	77
Retrospective	13	247/1035	521/1601	0.78	0.35–1.75	91
Setting						
ICU	12	161/564	450/977	0.53	0.30–0.93	72
Non-ICU	12	137/591	177/1041	1.44	0.65–3.22	85
ICU and non-ICU	2	294/1069	353/876	0.55	0.31–0.98	88
Duration of aPP						
< 6h	16	369/1298	608/1526	0.89	0.55–1.44	72
6–12 h	5	170/835	334/1019	0.33	0.06–1.87	97
> 12 h	3	43/116	92/206	0.54	0.16–1.78	79
Number of patients						
> 100	13	507/1970	903/2683	0.68	0.41–1.12	90
≤ 100	14	100/380	83/339	1.23	0.57–2.67	71
Duration of follow-up						
< 7 days	2	16/62	3/24	1.99	0.28–14.05	31
7–14 days	7	149/706	182/598	0.76	0.38–1.52	66
14–28 days	9	278/1055	622/1513	0.46	0.26–0.82	80
> 28 days	5	133/428	140/736	1.39	0.42–4.60	92
Period of study recruitment						
Before 31/12	20	498/1842	823/2418	0.75	0.65–0.87	86
After 31/12	5	33/323	79/396	0.48	0.30–0.76	41
Risk of bias						
High	8	339/1193	397/1546	1.29	0.59–2.81	90
Moderate	16	237/1077	521/1324	0.66	0.37–1.18	81
Low	3	31/80	68/165	0.95	0.52–1.72	0
DNR						
Included	3	237/703	259/1019	3.36	0.56–20.0	97
Excluded	6	307/995	441/1142	0.61	0.40–0.92	67
Not known	17	221/1054	490/1254	0.71	0.42–1.21	73

When the analysis was conducted by study design, awake prone positioning was associated with reduction of in-hospital death in retrospectives studies (18 studies, 3597 patients; 16.8% vs. 25.6%; random effect OR 0.45, 95% CI 0.30–0.66; *I*^2^ 57%) and not in prospective studies and in RCTs (Fig. 1; e-Fig. 4a); awake prone positioning was associated with reduction of orotracheal intubation in RCTs (six studies, 1934 patients; 28.5% vs. 34.0%; random effect OR 0.76, 95% CI 0.62–0.93; *I*^2^ 0%) and not in prospective or retrospective studies (Fig. 2; e-Fig. 4b).

In studies conducted in the ICU setting, awake prone positioning was associated with significant reduction of both in-hospital death (15 studies, 2397 patients; 18.2% vs. 23.3%; random effect OR 0.62, 95% CI 0.43–0.89, *I* ^2^ 36%) and orotracheal intubation (12 studies, 1541 patients; 28.5% vs. 46.0%; random effect OR 0.53, 95% CI 0.30–0.93; *I*^2^ 72%); in studies conducted outside the ICU setting or in mixed ICU or non-ICU patients, no association with mortality was observed (e-Fig. 5a significant reduction in orotracheal intubation was observed with awake prone positioning in studies reporting both ICU or non-ICU patients (two studies, 1945 patients; 27.5% vs. 40.2%; random effect OR 0.55, 95% CI 0.31–0.98; *I*^2^ 88%), while no effect was observed in studies conducted outside the ICU (Table 2; e-Fig. 6).

Concerning duration of awake prone positioning, a reduction in mortality was observed in studies using sessions lasting less than six hours or in studies using sessions lasting between 6 and 12 h, while no effect in mortality was observed in studies using awake prone positioning over 12 h (e-Fig. 7). No association was observed between duration of proning and orotracheal intubation (e-Fig. 8).

Awake prone positioning was associated with significant reduction in mortality in both studies including less than 100 patients and studies including more than 100 patients (Table 2; e-Fig. 9). No association was observed between the number of included patients and orotracheal intubation (e-Fig. 10).

A significant reduction of in-hospital mortality (9 studies, 2592 patients, 14.6% vs. 29.6%; random effect OR 0.26, 95% CI 0.11–0.64, *I*^2^ 86%) (e-Fig. 11) and of orotracheal intubation was observed in studies reporting on study outcome events occurring at 14–28 days ( 9 studies, 2568 patients, 26.3% vs. 41.1%; random effect OR 0.46, 95% CI 0.26–0.82, *I*^2^ 80%) (e-Fig. 12).

Awake prone positioning was associated with reduction in mortality in studies including patients until December 31, 2020, while this benefit disappeared in studies including patients beyond December 31, 2020 (e-Fig. 13). In addition, awake prone positioning was associated with a significant reduction in orotracheal intubation in studies including patients until December 31, 2020 (20 studies, 4260 patients; 27.0% vs. 34.0%; random effect OR 0.75, 95% CI 0.65–0.87, *I* ^2^ 86%) and beyond December 31, 2020 (five studies, 619 patients; 10.2% vs. 19.9%; random effect OR 0.48, 95% CI 0.30–0.76, *I*^2^ 41%)(e-Fig. 14).

Awake prone positioning was associated with a 50% reduction in mortality in studies with moderate risk of bias (19 studies, 3214 patients; 16.9% vs. 25.3%; random effect OR 0.50, 95% CI 0.33–0.75, *I*^2^ 56%), while no effect was observed in the remaining studies (e-Fig. 15). No association was observed with orotracheal intubation by quality of the studies (e-Fig. 16).

Of note, awake prone positioning was associated with a reduction in the risk of death and of orotracheal intubation in the 6 studies (2137 patients) that excluded DNR patients [17, 38, 39, 43, 48, 49].

## Discussion

Our systematic review and meta-analysis show that awake prone positioning reduces the composite of in-hospital death or orotracheal intubation in patients with acute respiratory failure due to COVID-19; in addition, our results suggest a reduced risk of in-hospital mortality by the use of awake prone positioning, mainly driven by retrospective studies and not observed in RCTs. Awake prone positioning was not associated with orotracheal intubation in the overall analysis, but it was in RCTs. The clinical benefits of awake prone positioning were more consistent for studies conducted in the setting of ICU in comparison to studies conducted outside the ICU.

Awake prone positioning has been proposed for the management of ARDS based on the hypothesis that the expected improvement in oxygenation would have led to an improvement in survival [51]. Reduction in mortality by prone positioning had been demonstrated in studies conducted with strict application of protective lung ventilation in non-COVID-19 intubated patients [52]. The COVID-19 outbreak led to a dramatic increase in the proportion of patients presenting with ARDS, probably due to the loss of hypoxic vasoconstriction leading to blood maldistribution and shunt effect. Prone positioning may improve ventilation/perfusion mismatch by increasing alveolar recruitment of basal regions and reduce dorsal over-distension. The high proportion of patients presenting with or progressing to ARDS during the COVID-19 outbreak led to change the use of awake prone positioning from a salvage therapy for refractory hypoxemia to an upfront lung-protective strategy intended to improve survival in severe respiratory failure. Of note, the benefit of medical treatments, if any, in improving clinical outcome in COVID-19 patients was dependent on early use, before progression to severe disease and in particular before the need for orotracheal intubation [53, 54]. For these reasons, the use of awake prone positioning was extended to awake patients in and out of ICUs, in an attempt to prevent disease progression [55].

Our meta-analysis shows that prone positioning reduces the composite of in-hospital death or orotracheal intubation. This composite outcome has been largely used in the COVID-19 era to assess the role of different treatment strategies. In fact, progression to orotracheal intubation was a common event and was strongly associated with mortality, mainly in the pre-vaccine era [56].

The analyses of aggregate data show a reduction in mortality by the use of awake prone positioning. This result is promising, as it suggests the effectiveness of a non-pharmacological management strategy in patients with acute hypoxemic respiratory failure. However, before encouraging a worldwide use of awake prone positioning in this setting, further evidence from high-quality studies is required. In fact, a reduction in mortality was not achieved in RCTs and is mainly driven by retrospective studies. Retrospective studies cannot be used to demonstrate the efficacy of clinical interventions as their results are likely to be influenced by confounding factors and the standardization of treatment is not guaranteed; in addition, in awake patients with acute hypoxemic respiratory failure [57], maintaining prone position can be particularly challenging and poor comfort has been reported mainly in those patients assisted by non-invasive ventilation. Finally, it should be noted that the use of prone positioning has been associated with delay in intubation, probably due to initial improvement in oxygenation. This delay can be harming for patients as delayed intubation increases work of breathing [58]and may increase mortality [59].

The analysis of aggregate data shows no significant effect of awake prone positioning in the risk for orotracheal intubation in all the studies; of note, a reduction in the risk for orotracheal intubation was observed in the analysis conducted in RCTs and in studies in patients managed in the ICU setting. It is conceivable that the use of standardized criteria for orotracheal intubation and the homogeneity of the study populations could have driven the results of the RCTs. In addition, a reduction in the risk for orotracheal intubation was observed in the analysis of studies that excluded DNR patients. Whether the failure to find a benefit in terms of progression to orotracheal intubation in the overall studies analysis is due to an actual limit of prone positioning or whether it is related to the high heterogeneity in study populations remains to be defined.

Concerning the sensitivity analyses, their results show that the association of awake prone positioning with the study outcomes substantially differ in RCTs in comparison to cohort studies; heterogeneity disappeared when the analysis was limited to RCTs. In addition, the benefits of awake prone positioning were mainly driven by studies conducted in the ICU setting, despite significant heterogeneity. Expertise in prone positioning could have driven the favorable results obtained in death and in orotracheal intubation in this setting. Despite no definite standardization exists on criteria for ICU admission, it is conceivable that COVID-19 patients admitted to ICU were probably more homogeneous in terms of severity of respiratory failure, reduced prevalence of DNR patients, and comorbidities respect to non-ICU patients. Sensitivity analysis by risk of bias showed persistence of significant heterogeneity regardless of the estimated risk of bias. Of note, the majority of the studies included in our meta-analysis had moderate risk of bias according to the Cochrane Collaboration’s Tools.

Our study has some limitations. Firstly, the intrinsic limit of the meta-analysis approach which combines heterogeneous datasets. This limit is particularly evident in our study, reporting a significant degree of heterogeneity in several analyses. In particular, the heterogeneity is lower when combining RCTs. It should be considered that our meta-analysis probably reports on first experience on prone positioning practiced outside the ICU and this may have required a time for acquisition of skillness and expertise. In addition, protocols of prone positioning were heterogeneous among studies, mainly concerning duration of cycles and duration of treatment, and this may have influenced the study results. Data in DNR patients and in vaccinated patients are not separately reported in the included studies and sensitivity analyses were not feasible. Furthermore, as these are aggregated data, meta-analysis with adjustment for age, comorbidities, severity of disease, or concomitant treatments was allowed. Finally, all included studies had an open design, that could have influenced decision making on orotracheal intubation.

Our study also has some strengths. The meta-analysis includes more than 30 studies, allowing analyses on over 5000 patients and the conduction of several sensitivity analyses that may generates hypotheses for future properly designed trials in this setting.

## Conclusion

Awake prone positioning reduces the risk for death or orotracheal intubation and, probably, the risk for death in patients with acute respiratory failure related to COVID-19, mainly when used in the setting of ICU. Further randomized studies should be conducted to confirm the clinical benefit of awake prone positioning and to validate standardized protocols for this procedure.

### Supplementary Information

Below is the link to the electronic supplementary material.

Supplementary file1 (DOCX 27445 KB)

## Data Availability

The database is available on reasonable request to the corresponding author.

## Abbreviations

aPP: Awake prone positioning
ARDS: Acute respiratory distress syndrome
COVID-19: COronaVIrus Disease-19
ICU: Intensive care unit
*P*/*F*: Partial pressure arterial oxygen and fraction of inspired oxygen
RR: Respiratory rate
