# Supplementary material for: Awake prone positioning for patients with COVID-19-related respiratory failure: a systematic review and meta-analysis
Source: Intern Emerg Med. 2023 Oct 5;19(1):147–58. doi: 10.1007/s11739-023-03434-1 (PMC10827908; doi:10.1007/s11739-023-03434-1)

Supplementary Material

e-Appendix 1. Search strategy

e-table 1. Baseline characteristics of the studies included in the meta-analysis

e-table 2. Cochrane Collaboration’s tool to assess risk of bias in randomized trials (RoB 2)

e-table 3. Cochrane Collaboration’s tool to assess risk of bias in non-randomized studies of interventions. (Robins-1)

e-figure 1. PRISMA 2020 - flow diagram

e-figure 2. In-hospital death (correction for zero cells was performed by adding 0.5)

e-figure 3. Orotracheal intubation (correction for zero cells was performed by adding 0.5)

e-figure 4. Funnel plot In-hospital death (panel a) and funnel plot orotracheal intubation (panel b)

e-figure 5. Forest plot and funnel plot in-hospital death by setting

e-figure 6. Forest plot and funnel plot orotracheal intubation by setting

e-figure 7. Forest plot and funnel plot in-hospital death by duration of aPP

e-figure 8. Forest plot and funnel plot orotracheal intubation by duration of aPP

e-figure 9. Forest plot and funnel plot in-hospital death by patients included in the studies

e-figure 10. Forest plot and funnel plot orotracheal intubation by patients included in the studies

e-figure 11. Forest plot and funnel plot in-hospital death by duration of follow-up

e-figure 12. Forest plot and funnel plot orotracheal intubation by duration of follow-up

e-figure 13. Forest plot and funnel plot in-hospital death by period of study recruitment

e-figure 14. Forest plot and funnel plot orotracheal intubation by period of study recruitment

e-figure 15. Forest plot and funnel plot in-hospital death by quality of the studies

e-figure 16. Forest plot and funnel plot orotracheal intubation by quality of the studies

**e-Appendix 1. Search strategy**

| OVID: COVID* and pron*  WoS: Coronavirus OR COVID* AND pron*  Pubmed: coronavirus OR COVID* and pron* |
| --- |
| <https://clinicaltrials.gov/search?cond=COVID-19&term=Prone%20Positioning> |
| <https://www.medrxiv.org/search/covid-19%252BAND%252Bpron%252A%20numresults%3A10%20sort%3Apublication-date%20direction%3Adescending> |

e-table 1. Baseline characteristics of the studies included in the meta-analysis

|  | **Study design** | **Setting** | **Patients (n)** | **Male (%)** | **Age, years** | **Definition of**  **Severity of respiratory failure** | **Follow-up days** | **Use of NIV (CPAP or PSV)** | **Use of HFNC** | **Treated by aPP**  **n/N**  **(%)** | **Predefined protocol for aPP (duration of cycles, duration)** |
| --- | --- | --- | --- | --- | --- | --- | --- | --- | --- | --- | --- |
| Alhazzani W.  2022 ^17^ | RCT | ICU | 400 | 28 | 57 | P/F | 60 | - | - | 205/400  (51.25) | 2-8 h, 4 days |
| Altinay M.  2021^18^ | R | ICU | 72 | 28 | 67 | P/F | 28 | - | - | 25/72  (34.72) | 12-18h,- |
| Ates I.  2021^19^ | R | Non-ICU | 144 | 72 | 60 | O2 Suppl. | 15 | 5 | 4 | 97/144 (67.36) | 6-8h,5-12 days |
| Bahloul M. 2021^20^ | P | ICU | 96 | 60 | 61 | O2 Suppl. | n.a. | - | 16 | 21/96 (21.87) | 2-4h |
| Barker J.  2021^21^ | R | ICU | 20 | 60 | 62 | P/F | 30 | 18 | - | 10/20 (50.00) | 30’-2h,7 days |
| Burton-Papp HC.  2020^22^ | R | ICU | 81 | 54 | 54 | P/F | 10 | 20 | - | 20/81 (22.98) | 1-6h,- |
| Coppo A.  2020^23^ | P | Non-ICU | 56 | 79 | 57 | O2 Suppl. | 5 | 56 | - | 47/56 (83.92) | 3-8h,5days |
| Ehrmann S.  2021^24^ | RTCs | ICU  Non-ICU | 1121 | 746 | 60.5 | P/spO2 | 28 |  | 1121 | 564/1121  (50.3) | 3h,- |
| Ferrando C. 2020^25^ | P | ICU | 199 | 74 | 64 | Berlin  criteria | 28 | - | 199 | 55/199 (27.63) | 16h,- |
| Fralick M.  2022^26^ | RCT | Non-ICU | 248 |  | 56 | O2 Suppl. | 28 | 11 | - | 126/248 (50.80) | 2h,3 days |
| Gad S.  2021^27^ | P | ICU | 30 | 57 | 47 | O2 Suppl. | 3 | 15 | - | 15/30 (50.00) | 3h,- |
| Graziani M.  2023^28^ | R | Non-ICU | 536 | 334 | 69 | Berlin criteria | 30 | 187 | 46 | 114/536  (21.2) | 4-10h, 3 times a day |
| Hallifax RJ.  2020^29^ | R | Non- ICU | 48 | 67 | 69 | O2 Suppl. | n.a. | - | 26 | 30/48 (62.50) | 2h for twice a day, 2 days |
| Hashemian  SM.  2021^30^ | P | ICU | 75 | 69 | 65 | Berlin  criteria | 14 | 75 | - | 45/75 (60.00) | 30’ every 4h |
| Hussain HT.  2021^31^ | R | ICU | 50 | 80 | 50 | RR | 15 | - | - | 25/50 (50.00) | 7h,- |
| Imran M.  2021^32^ | R | ICU | 100 | 76 | 51 | O2 Suppl. | 14 | - | - | 50/100 (50.00) | 5-6h,- |
| Jagan N.  2020^33^ | R | ICU | 105 |  | 60 | O2 Suppl. | 14 | - | - | 40/105 (38.09) | - |
| Jayakumar D. 2021^34^ | RCT | Non-ICU | 60 | 83 | 55 | P/F | 7 | 5 | 15 | 30/60 (50.00) | 6h,- |
| Johnson SA. 2021^35^ | RCT | Non-ICU | 30 | 53 | 62 | P/F | 72h | 3 | 2 | 15/30 (50.00) | 1.5h,- |
| Jouffroy R.  2021^36^ | R | ICU | 379 | 77 | 61 | P/F | 28 | 27 | 146 | 40/379 (10.55) | 3-6h,3 days |
| Liu X.^37^  2020 | R | Non-ICU | 29 | 79 | 45 | O2 Suppl. | 14 | - | - | 13/29 (44.82) | 10-14h,14 days |
| Musso G.^38^  2022 | P | Non-ICU | 243 | 178 | 68 | P/F | 28 | 243 | - | 81/243  (33.3) | 8 h,- |
| Padrao EMH. 2020^39^ | R | Non-ICU | 166 | 68 | 58 | O2 Suppl. | 15 | - | - | 57/166 (34.33) | 30’-4h,15 days |
| Perez-Nieto OR.  2021^40^ | R | ICu-non-ICU | 827 | 73 | 54 | P/F | 10 | - | 83 | 505/827 (61.06) | 12h,- |
| Proud'homme E.  2021^41^ | R | Non- ICU | 178 | 38 | 62 | O2 Suppl. | 14 | - | - | 48/178 (26.96) | 3-12h,3 days |
| Qian  ET.  2022^42^ | P | Non-ICU | 501 | 56 | 61 | - | 20h | 35 | 133 | 239/501 (47.70) | - |
| Rosen J.  2021^43^ | RCT | Non- ICU | 75 | 73 | 65 | P/F | 30 | - | 60 | 36/75 (48.00) | 16h,- |
| Simioli F.  2021^44^ | R | Non-ICU | 29 |  | 66 | P/F | 14 | - | 6 | 18/29 (62.06) | 2h,- |
| Syrma PB.  2021^45^ | P | Non-ICU | 45 | 84 | 53 | O2 Suppl. | 20h | 2 | 1 | 30/45 (66.66) | 7,5h,- |
| Stilma W.  2021^46^ | R | ICU | 734 |  | 64 | P/F | 12h | 734 | - | 438/734 (59.67) | 10h,1 day |
| Thompson A. 2020^47^ | P | Non-ICU | 29 | 76 | 67 | RR | 1h | - | - | 25/29 (86.20) | 1h,- |
| Tonelli R 2021^48^ | R | ICU | 114 | 70 | 67 | P/F | n.a. | 19 | 69 | 38/114 (33.33) | 3-12h,- |
| Vianello A.  2021^49^ | P | ICU | 93 | 63 | 68 | O2 Suppl. | 101 | 22 | 183 | 50/93 (53.76) | 2-4h,- |
| Zang X.  2020^50^ | R | Non-ICU | 60 |  | 64 | P/F | 90 | 15 | 15 | 23/60 (38.33) | - |

Abbreviations: CPAP: Continuous Positive Airway Pressure; HFNC: high flow nasal cannula; NIV: non-invasive mechanical ventilation; PSV: pressure support ventilation; P/F: partial pressure arterial oxygen and fraction of inspired oxygen (PaO2/FiO2 ratio); PP prone positioning;

RR: respiratory rate; ICU: intensive care unit

e-Table 2. Cochrane Collaboration’s tool to assess risk of bias in randomized trials (RoB 2)

| **Author,year** | **Random sequence generation** | **Allocation concealment** | **Blinding of partecipants and personnel** | **Blinding of outcome assessment** | **Incomplete outcome data** | **Selective reporting** | **Others bias** | **Summery**  **assessments** |
| --- | --- | --- | --- | --- | --- | --- | --- | --- |
| Alhazzani W.  2022^17^ | Low risk | Low risk | Low risk * | Low risk * | Low Risk | Low Risk | ? | Low risk of bias |
| Ehrmann S.  2021^24^ | Low risk | Low risk | Low risk * | Low risk * | Low risk | Low risk | ? | Low risk of bias |
| Fralick M.  2022^26^ | Low risk | Low risk | Low risk * | Low risk * | Low risk | Low Risk | ? | Low risk of bias |
| Johnson SA.  2021^35^ | Some concerns | Low risk | Low risk * | Low risk * | Low Risk | Low Risk | ? | Moderate risk of bias |
| Jayakumar D.  2021^34^ | Low risk | Some concerns | Low risk * | Low risk * | Low Risk | Low Risk | ? | Moderate  Risk of bias |
| Rosen J.  2021^43^ | Low risk | Low risk | Low risk * | Low risk * | Some concerns | Low Risk | ? | Low risk of bias |

*The nature of the intervention precluded blinding of participating patients

| Author, Year | Bias Due To Confounding | Bias In Selection Of Participants Into The Study | Bias In Classification Of Interventions | Bias Due To Deviations From Intended Interventions | Bias Due To Missing Data | Bias In Measurement Of Outcomes | Bias In Selection Of The Reported Result | Overall Bias |
| --- | --- | --- | --- | --- | --- | --- | --- | --- |
| Altinay M.2021^18^ | L | L | L | L | M | L | M | MODERATE |
| Ates I. 2021^19^ | M | L | M | L | M | L | M | MODERATE |
| Bahloul M. 2021^20^ | L | L | L | L | M | L | M | MODERATE |
| Barker J. 2021^21^ | L | L | M | S | L | L | M | SERIOUS |
| Burton-Papp H. 2020^22^ | L | L | L | L | L | L | L | LOW |
| Coppo A. 2020^23^ | L | L | L | L | L | L | L | LOW |
| Ferrando C. 2020^25^ | L | L | S | L | M | L | S | CRITICAL |
| Graziani M.2023^28^ | L | L | L | L | L | L | L | LOW |
| Gad S. 2021^27^ | L | L | S | L | L | L | S | CRITICAL |
| Hallifax RJ.2020^29^ | L | L | L | S | M | L | S | SERIOUS |
| Hashemian SM. 2021^30^ | L | L | L | L | L | L | M | MODERATE |
| Hussain HT. 2021^31^ | L | L | L | L | M | L | M | MODERATE |
| Imran M. 2021^32^ | L | L | S | L | M | M | M | SERIOUS |
| Jagan N. 2020^33^ | L | L | L | L | M | L | L | MODERATE |
| Jouffroy R.  2021^36^ | L | L | M | L | L | M | L | MODERATE |
| Liu X. 2020^37^ | L | L | L | L | L | M | L | MODERATE |
| Musso G. 2023^38^ | L | L | L | L | L | L | L | LOW |
| Padrao EMH.  2020^39^ | L | L | L | L | M | M | L | MODERATE |
| Perez-Nieto Or.  2021^40^ | L | L | L | L | M | L | L | MODERATE |
| Proud'homme E. 2021^41^ | L | M | L | L | M | M | L | MODERATE |
| Qian ED. 2022^42^ | L | L | L | L | L | L | L | LOW |
| Simioli F.  2021^44^ | L | L | M | L | M | L | M | MODERATE |
| Syrma PB. 2021^45^ | L | L | L | L | M | S | L | SERIOUS |
| Stilma W.  2021^46^ | L | L | L | L | M | L | L | MODERATE |
| Thompson A.  2020^47^ | L | L | L | L | M | M | L | MODERATE |
| Tonelli R.  2021^48^ | L | L | L | L | L | L | M | MODERATE |
| Vianello A. 2021^49^ | L | L | L | L | M | M | L | MODERATE |
| Zang X. 2019^50^ | L | L | L | L | L | L | M | MODERATE |

e-Table 3. Cochrane Collaboration’s tool to assess risk of bias in non-randomized studies of interventions. (Robins-1)

Abbreviations: L: low; M: moderate; S: serious

e-Figure 1. PRISMA 2020 - flow diagram

**Identification of studies via databases and registers**

4933from MEDLINE

3408 from WoS

1102 from Medrxiv

**Identification**

Records excluded:8676

Exclusion criteria:

Not on humans 457

No COVID-19 patients 852

Systematic Reviews,Letters, case series,case report 2601

Number of patients < 20 956

No prone positioning as intervention 3810

Records screened: 9443

Reports not retrieved: 656

Non reporting on study outcomes 200

Including intubated patients 310

No PCR confirmation for SARS-CoV2 146

Reports sought for retrieval:767

**Screening**

Reports excluded:

Duplication 45

No comparison between proning and supine 32

Reports assessed for eligibility: 111

Studies included in review: 34

**Included**

e-figure 2. In-hospital death (correction for zero cells was performed by adding 0.5

**
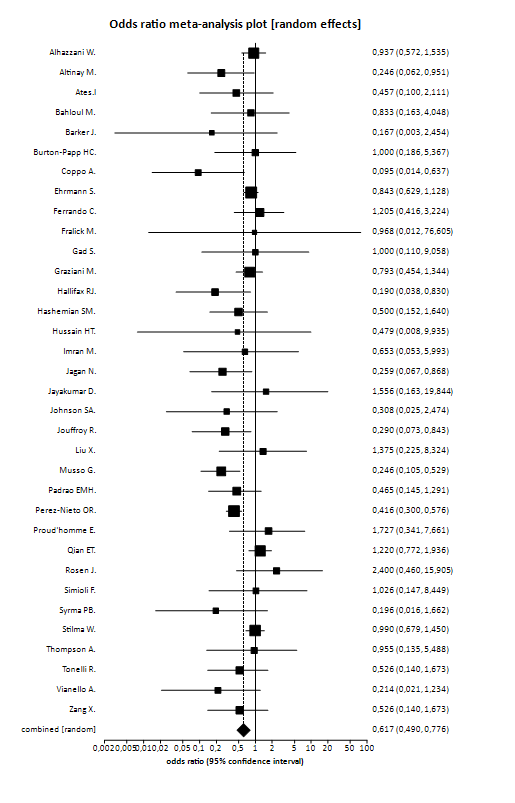
**

e-figure 3. Orotracheal intubation (correction for zero cells was performed by adding 0.5)


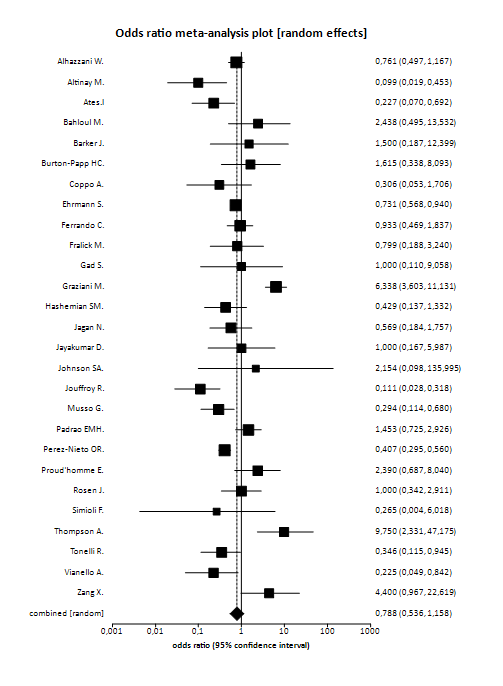


e-figure 4. Funnel plot In-hospital death (panel a) and funnel plot orotracheal intubation (panel b)

Panel a Panel b
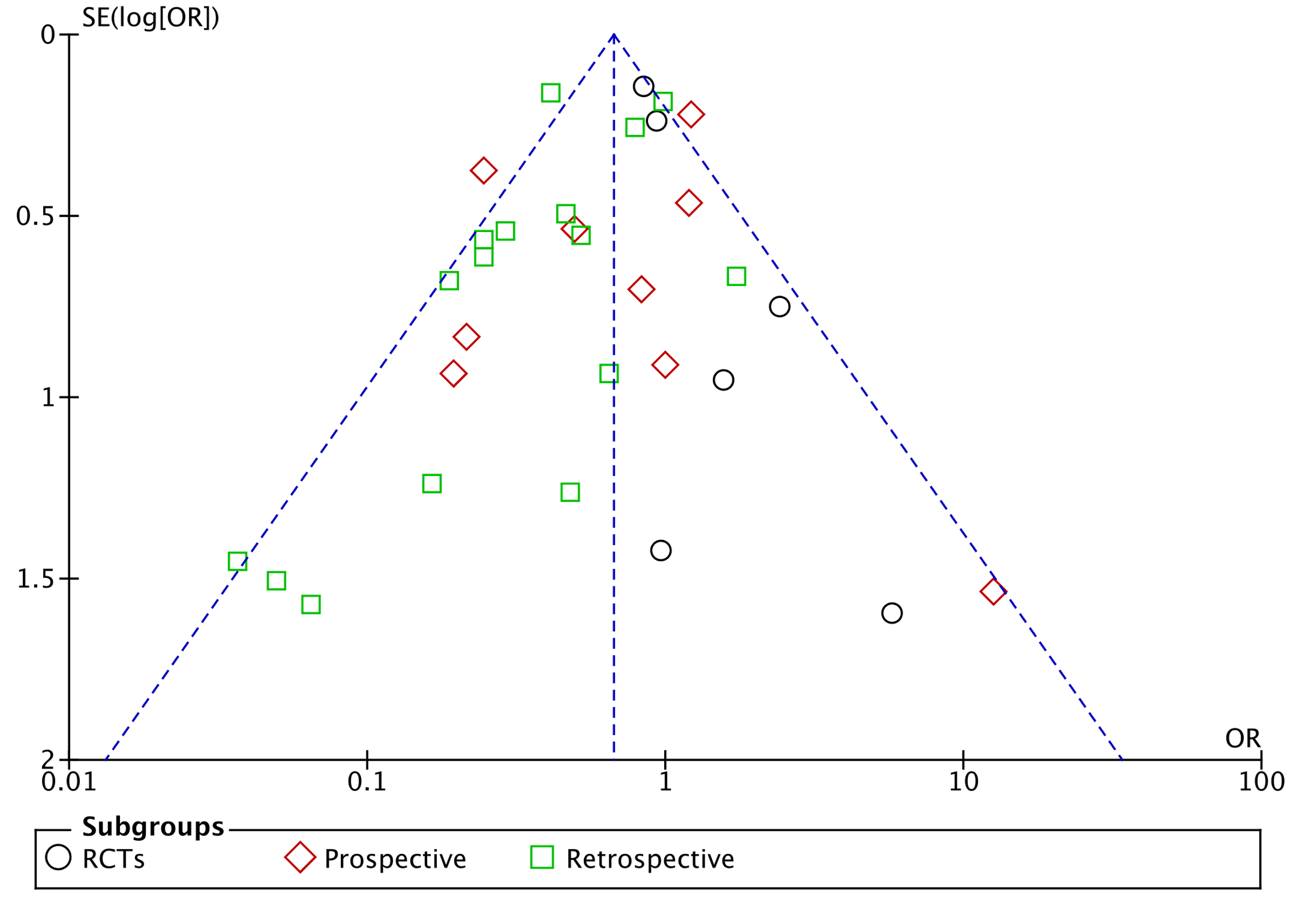

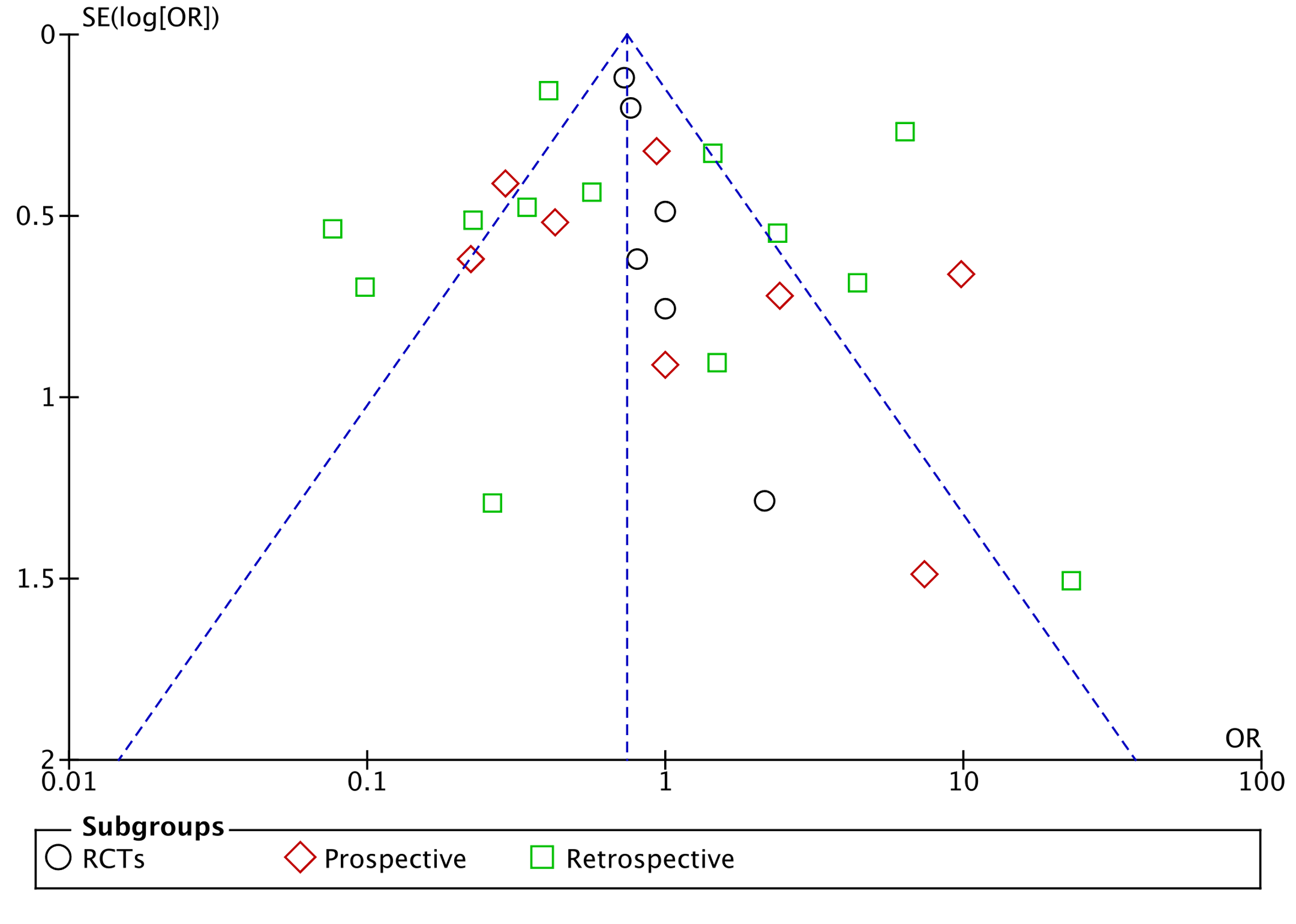


e-figure 5 Forest plot and funnel plot in-hospital death by setting


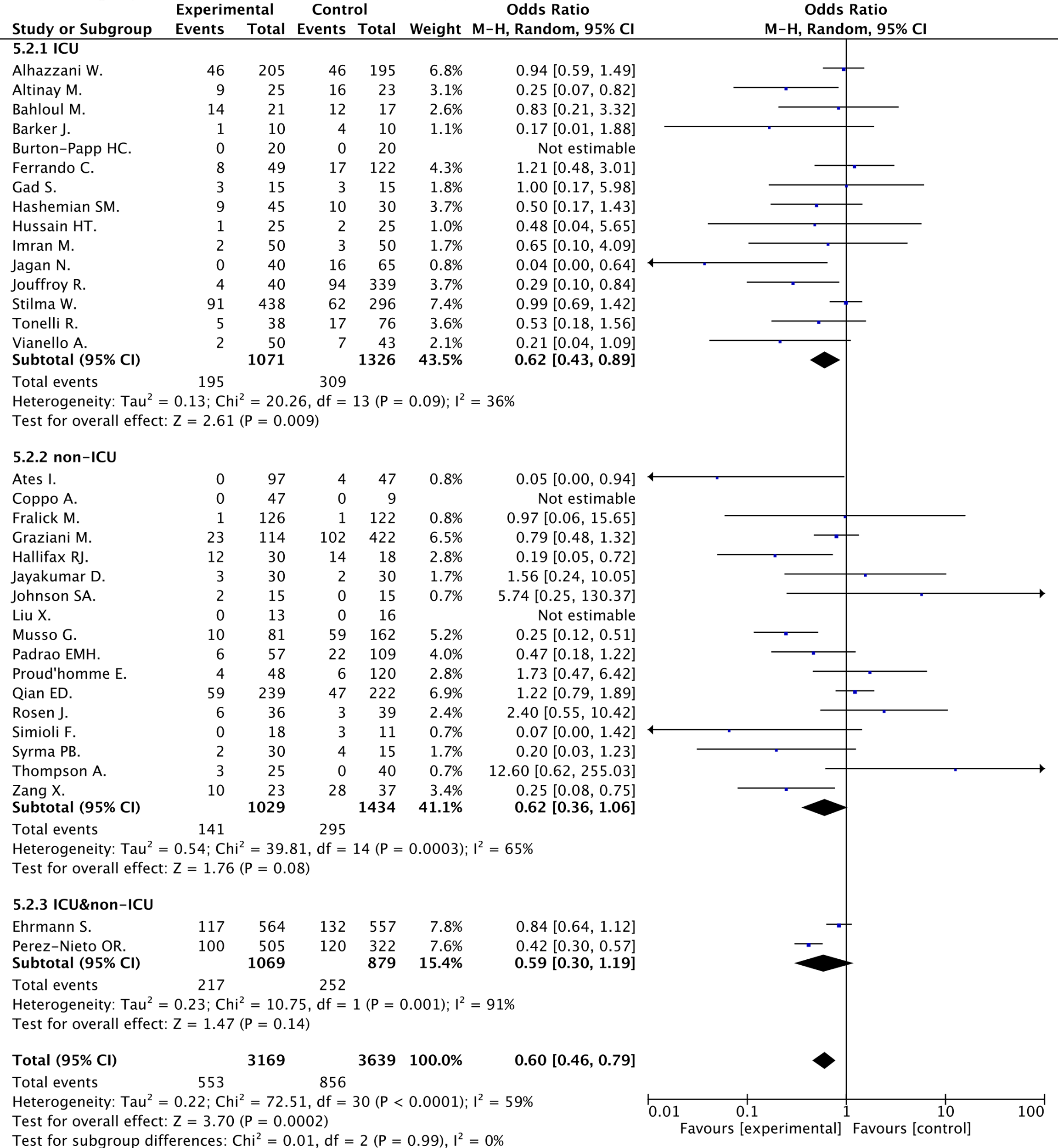

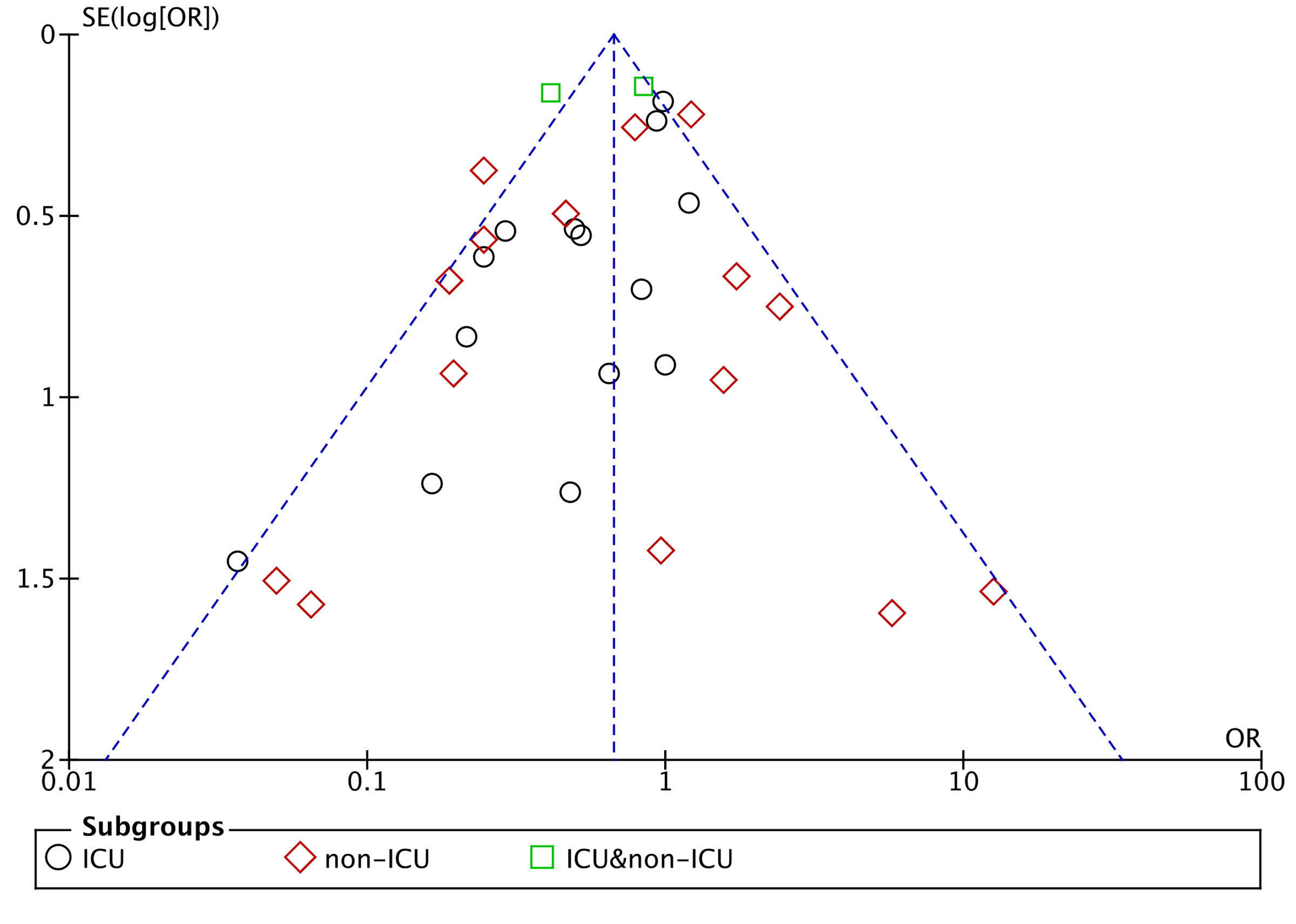


e-figure 6 Forest plot and funnel plot orotracheal intubation by setting


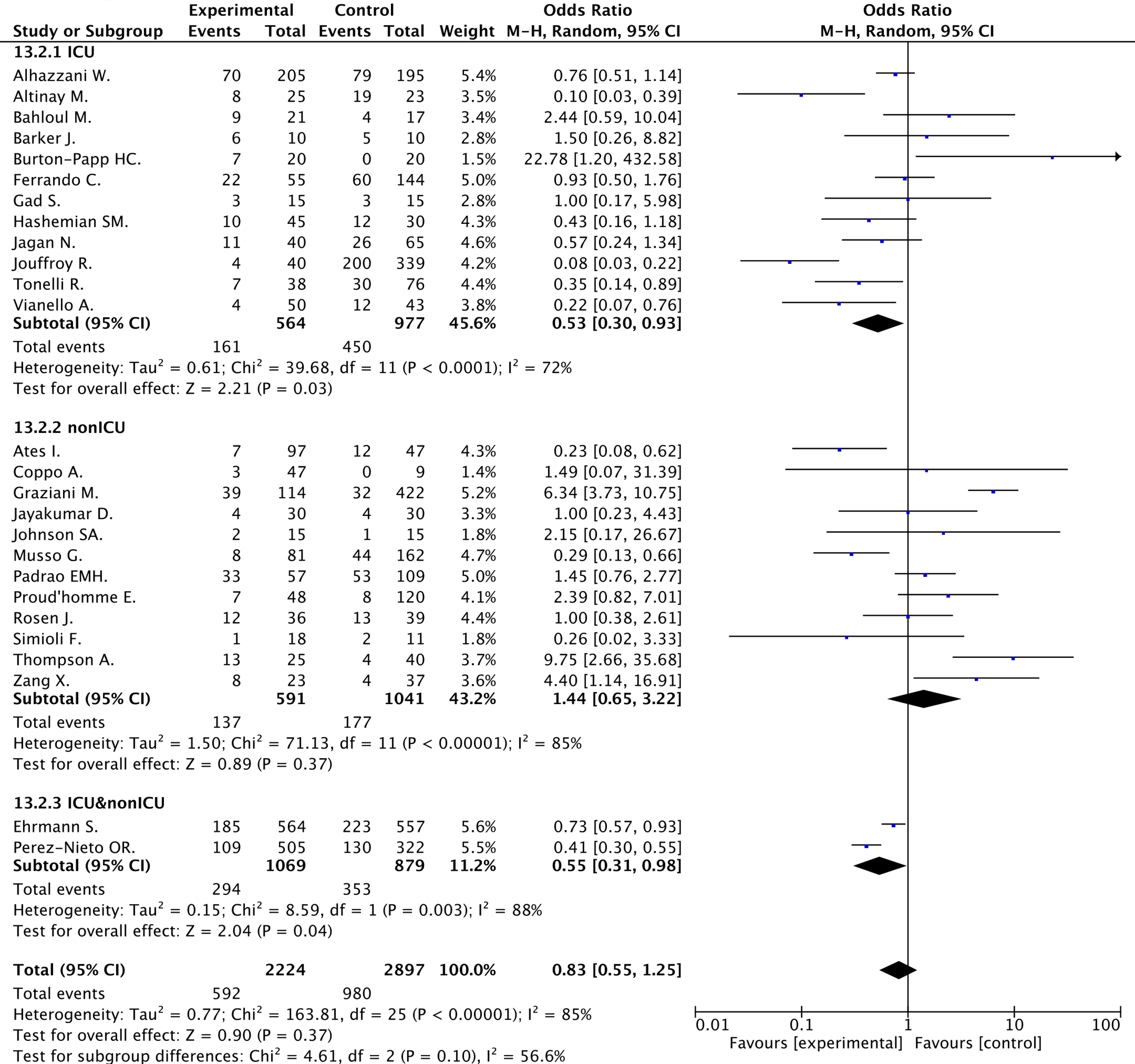


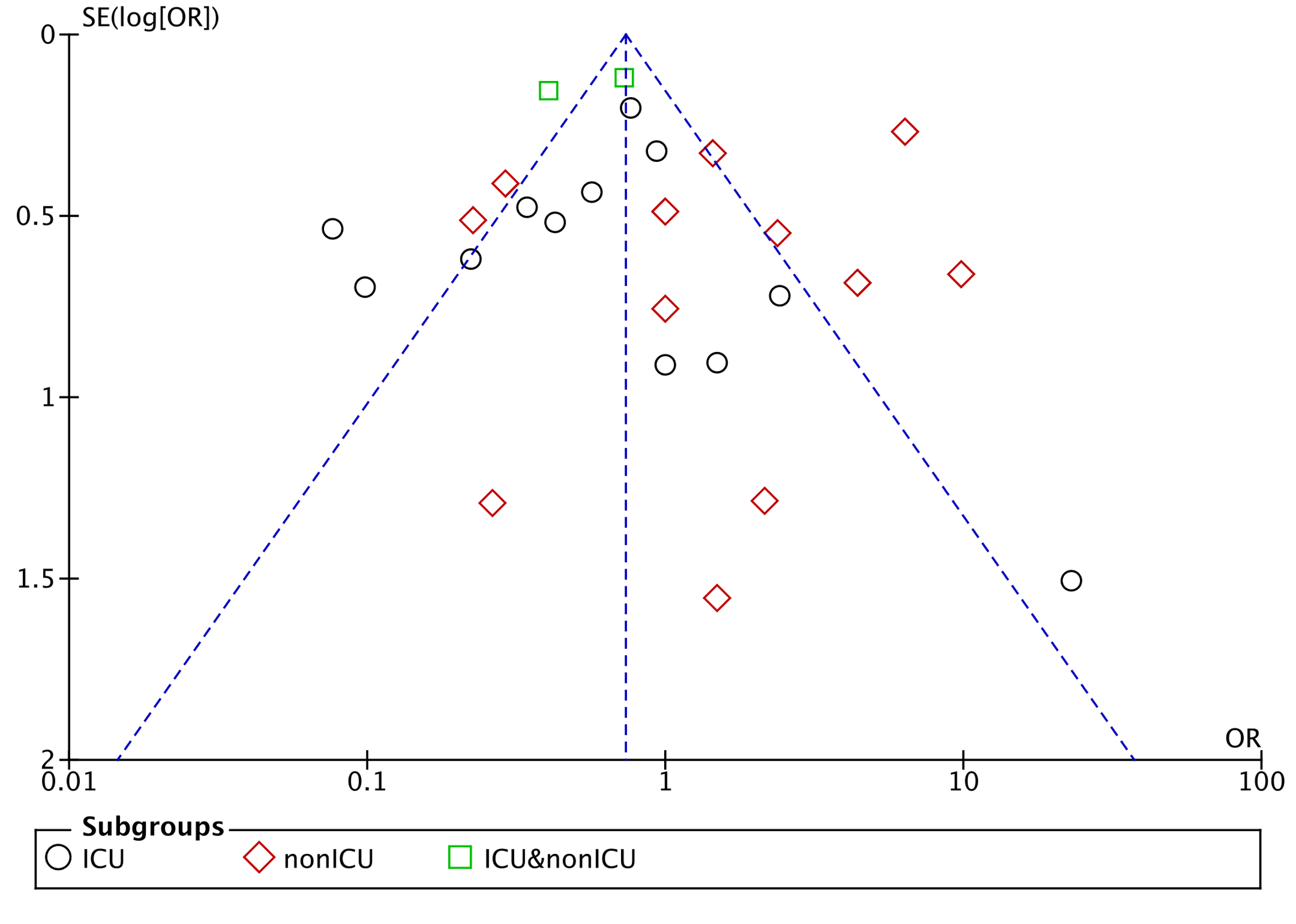


e-figure 7. Forest plot and funnel plot in-hospital death by duration of aPP


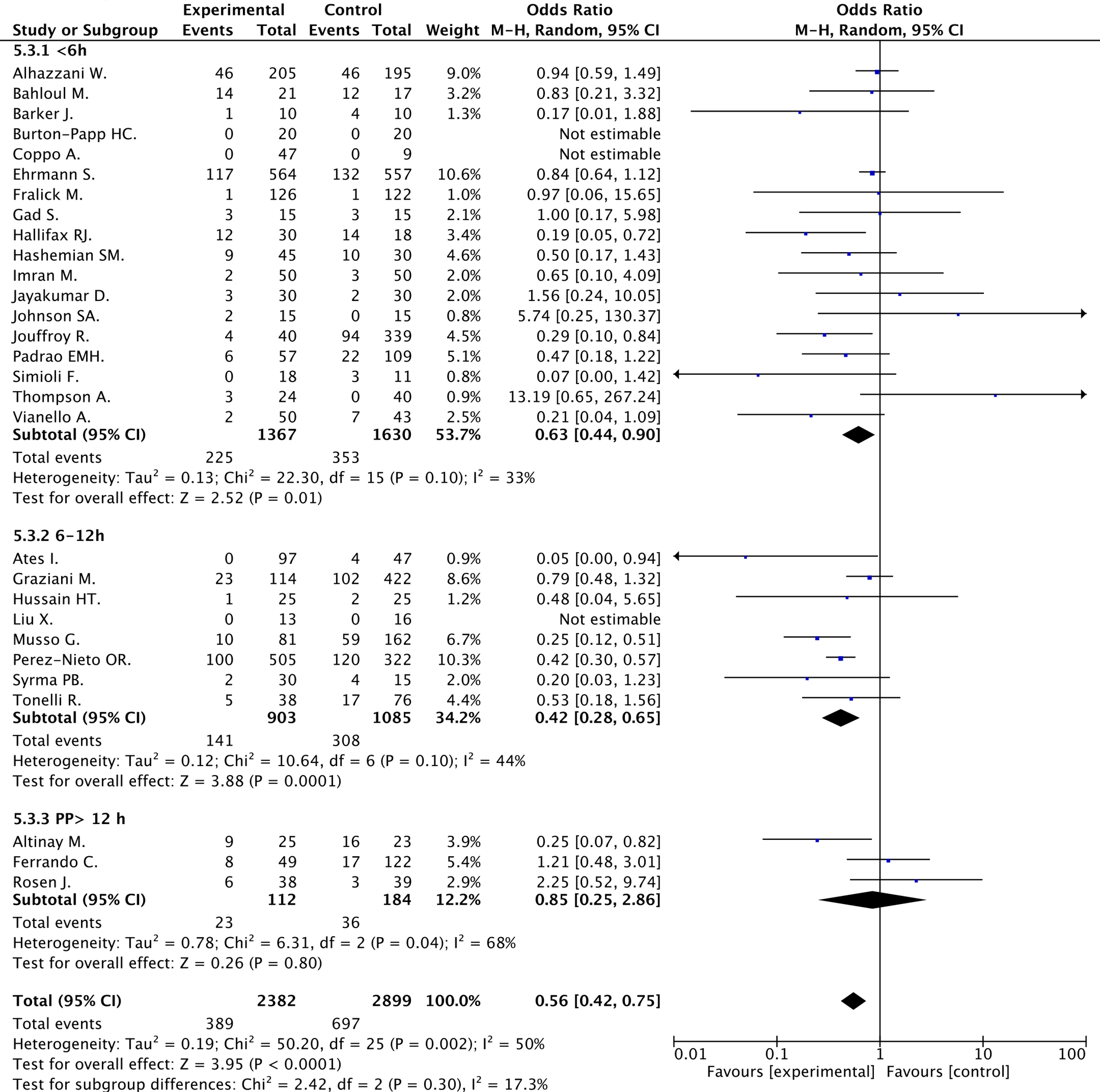

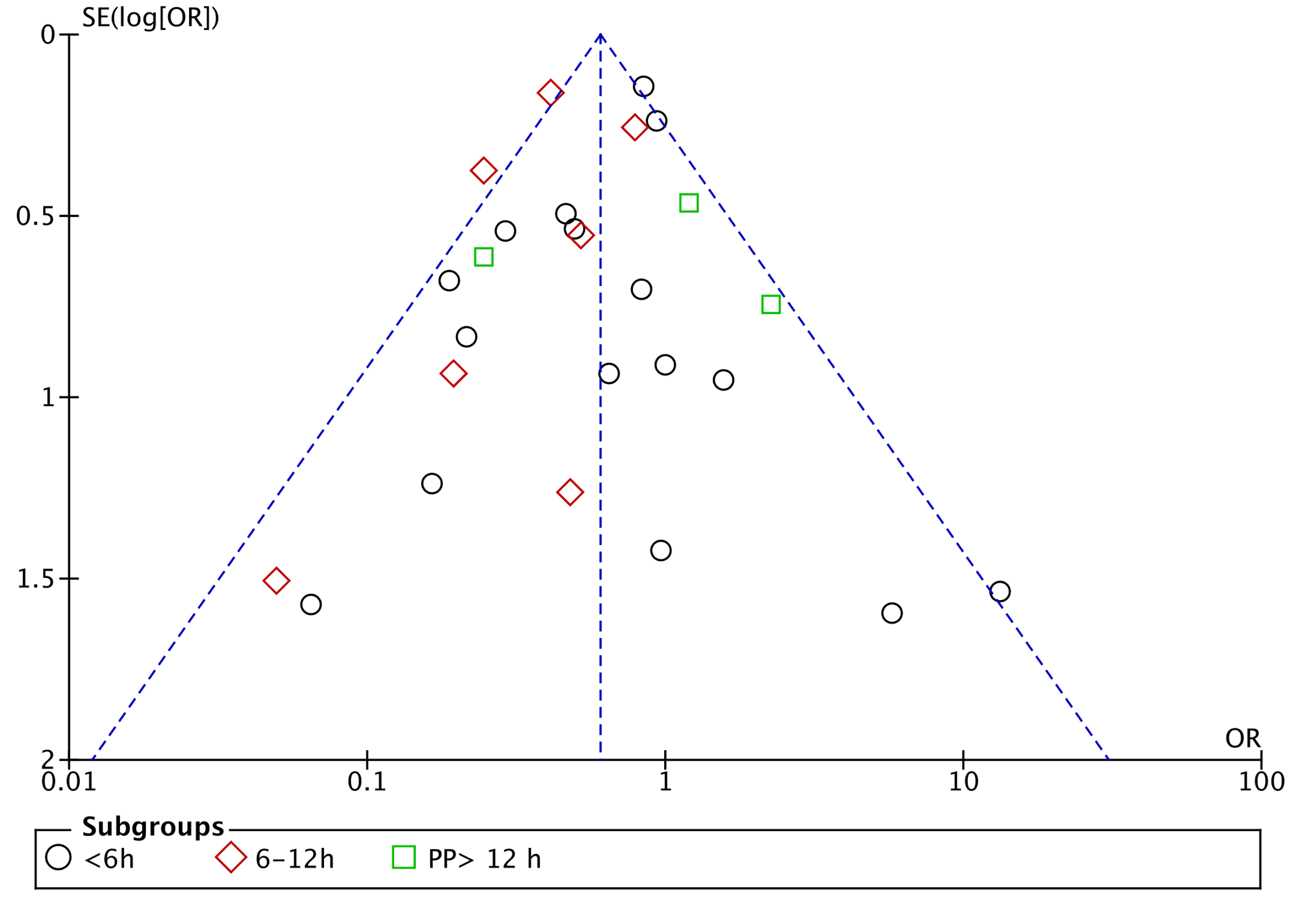


e-figure 8. Forest plot and funnel plot orotracheal intubation by duration of aPP


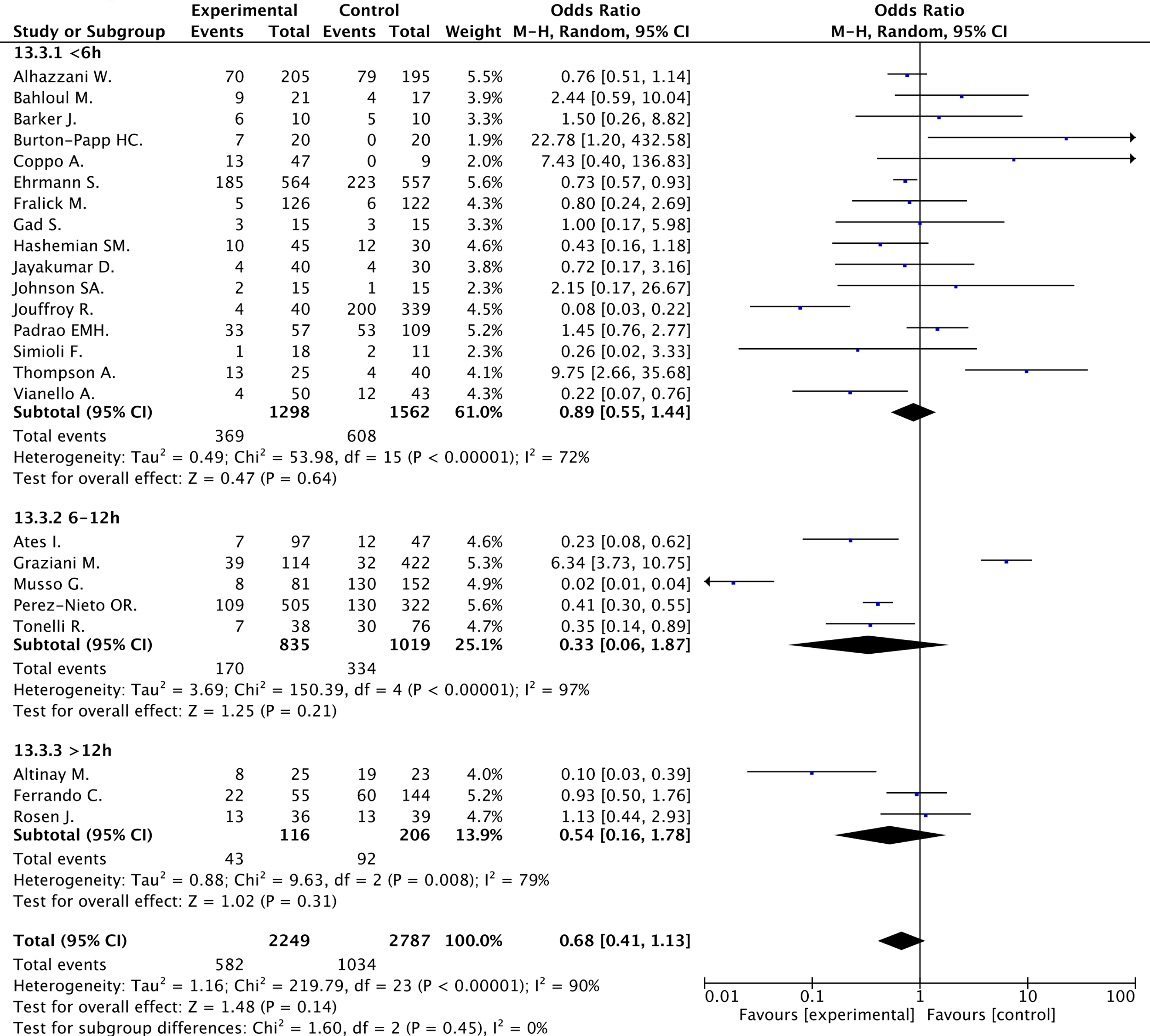

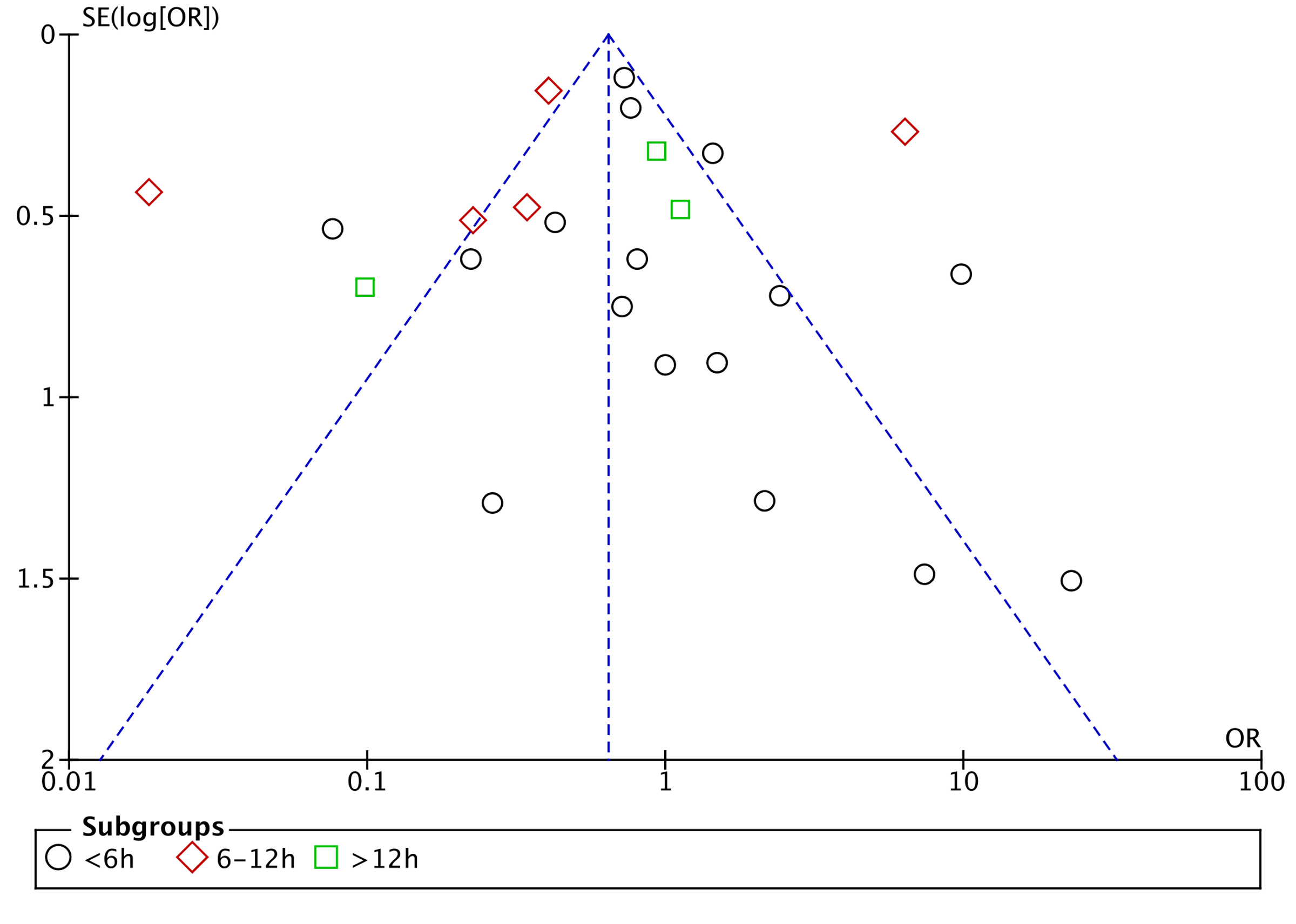


e-figure 9. Forest plot and funnel plot in hospital-death by patients included in the studies


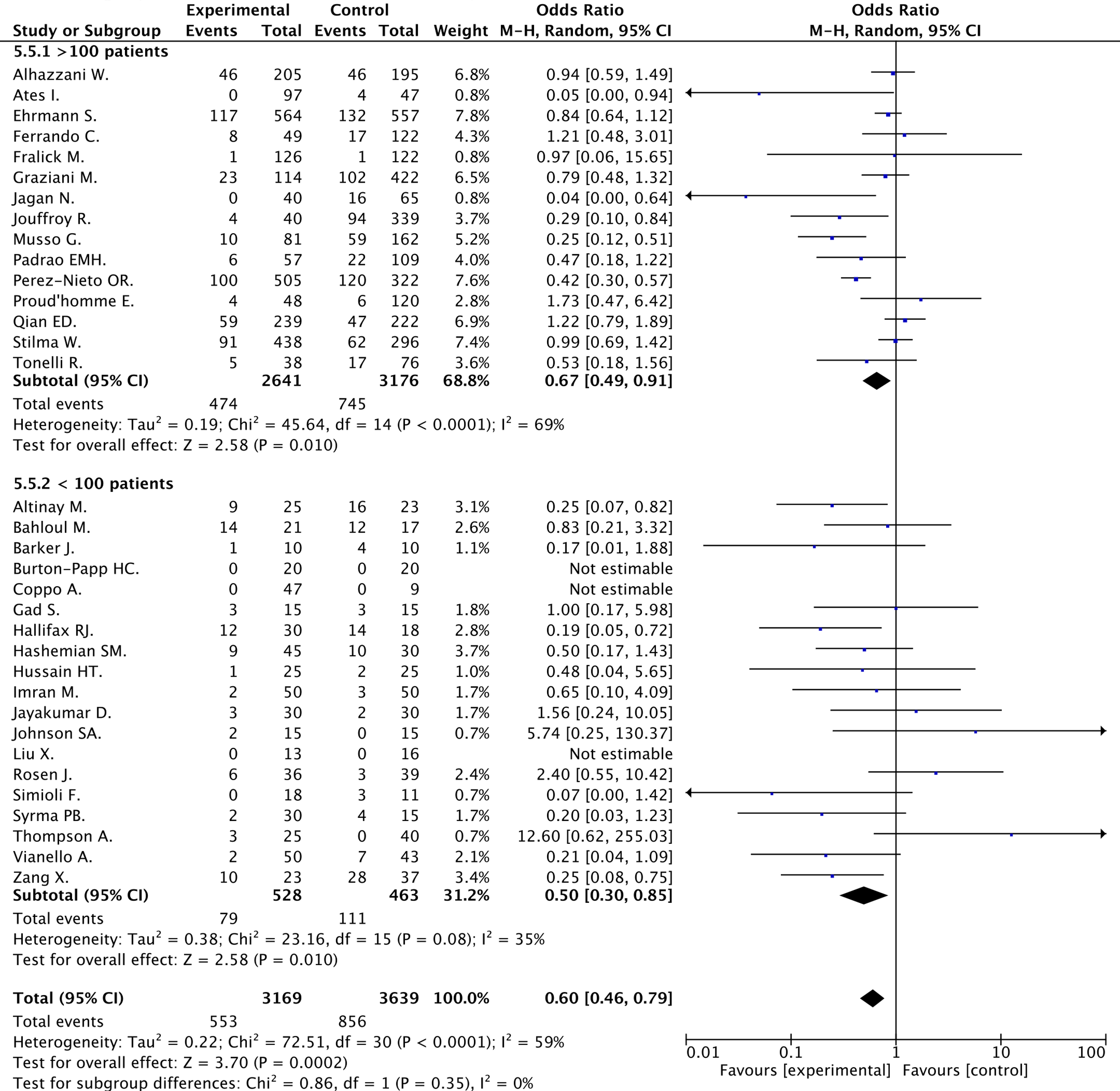

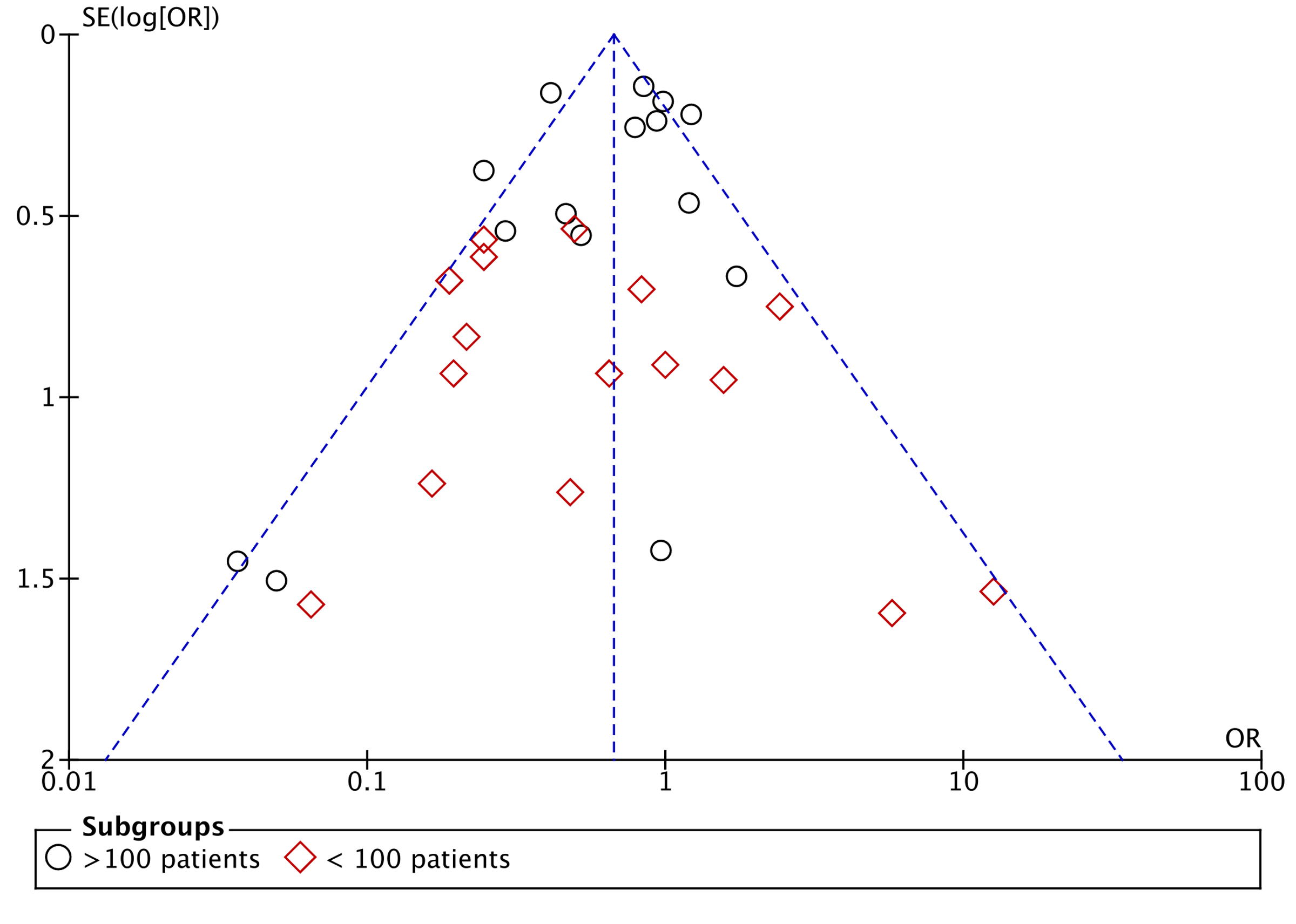


e-figure 10. Forest plot and funnel plot orotracheal intubation by patients included in the studies


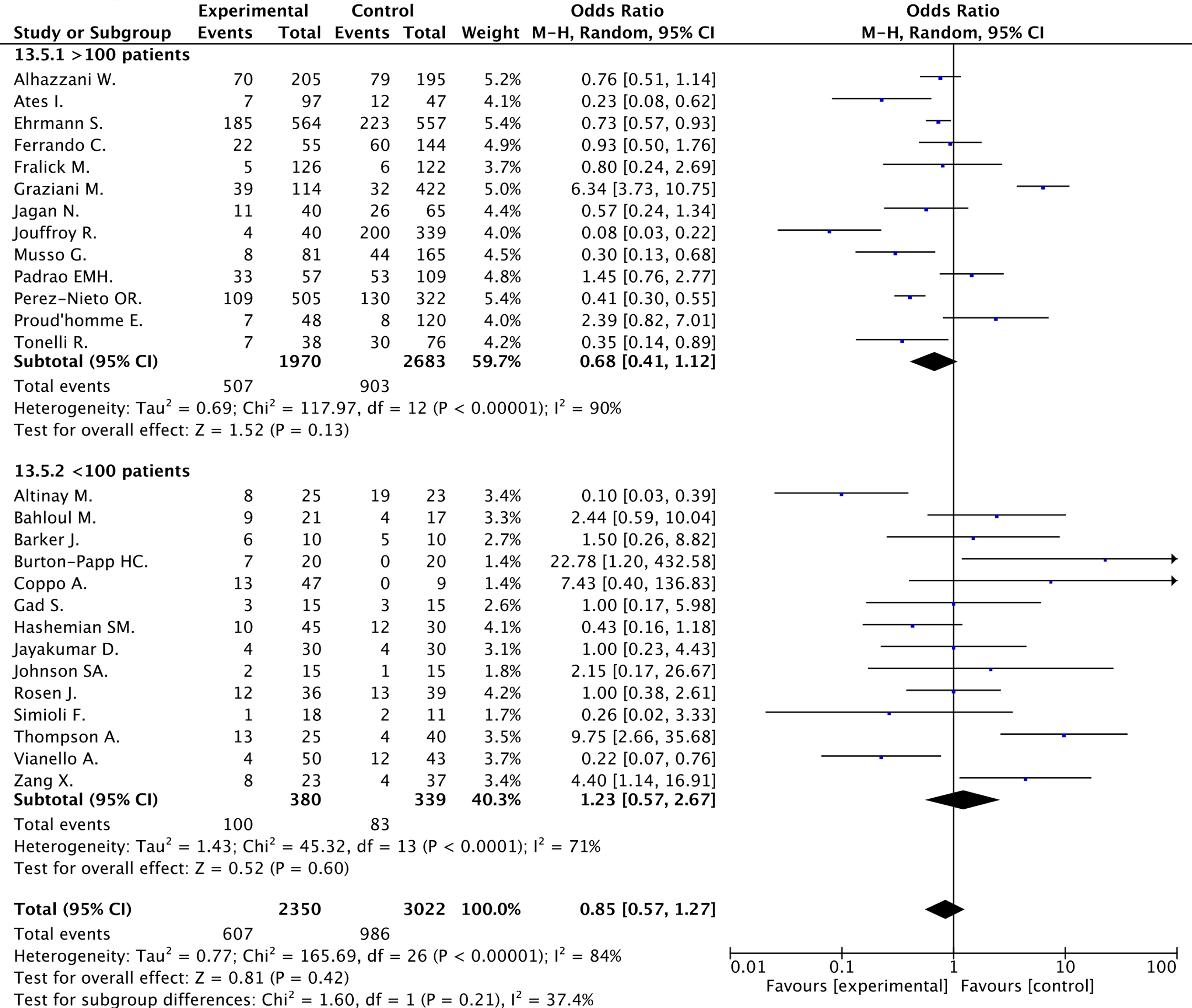

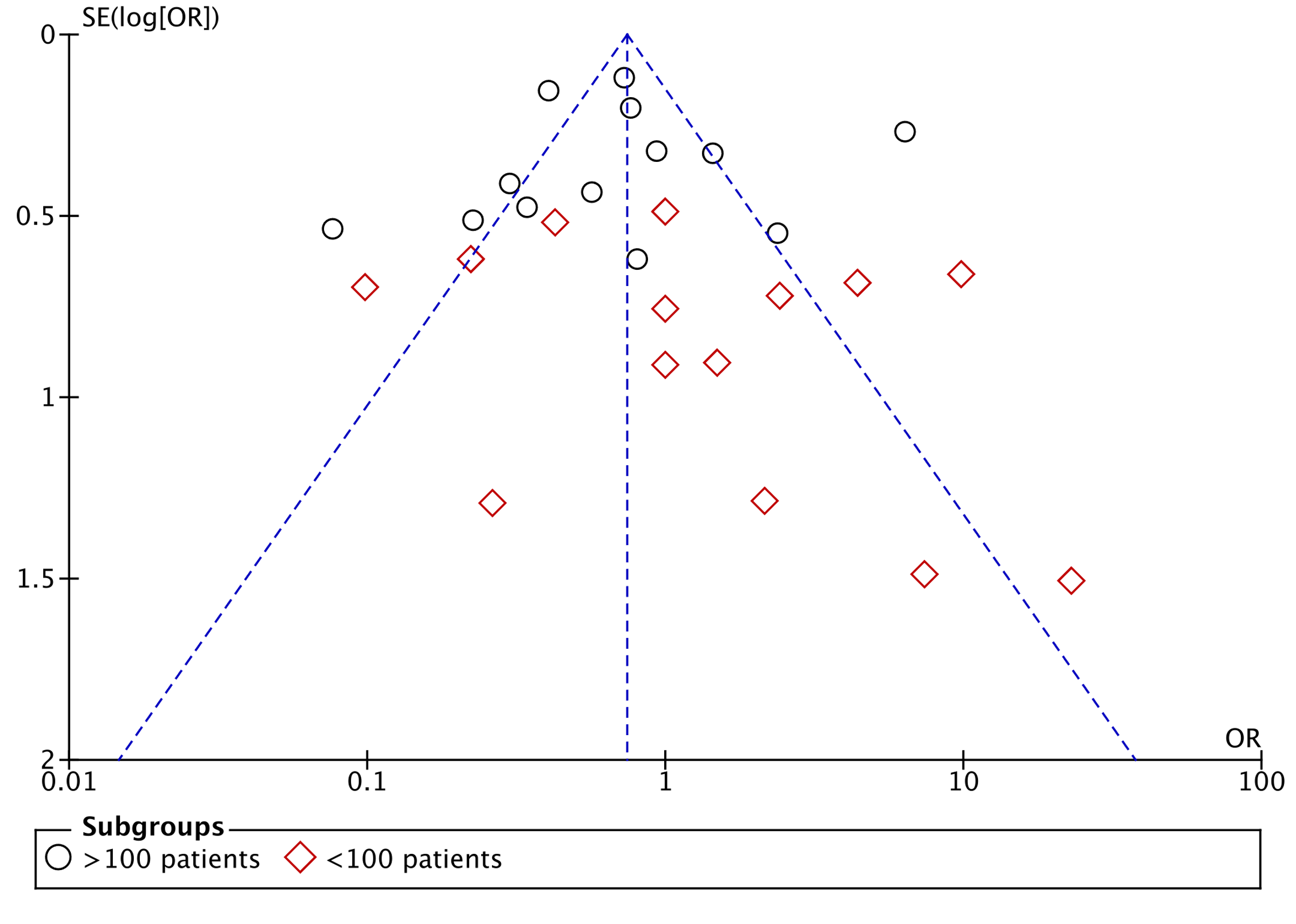


e-figure 11. Forest plot and funnel plot in hospital-death by duration of follow-up


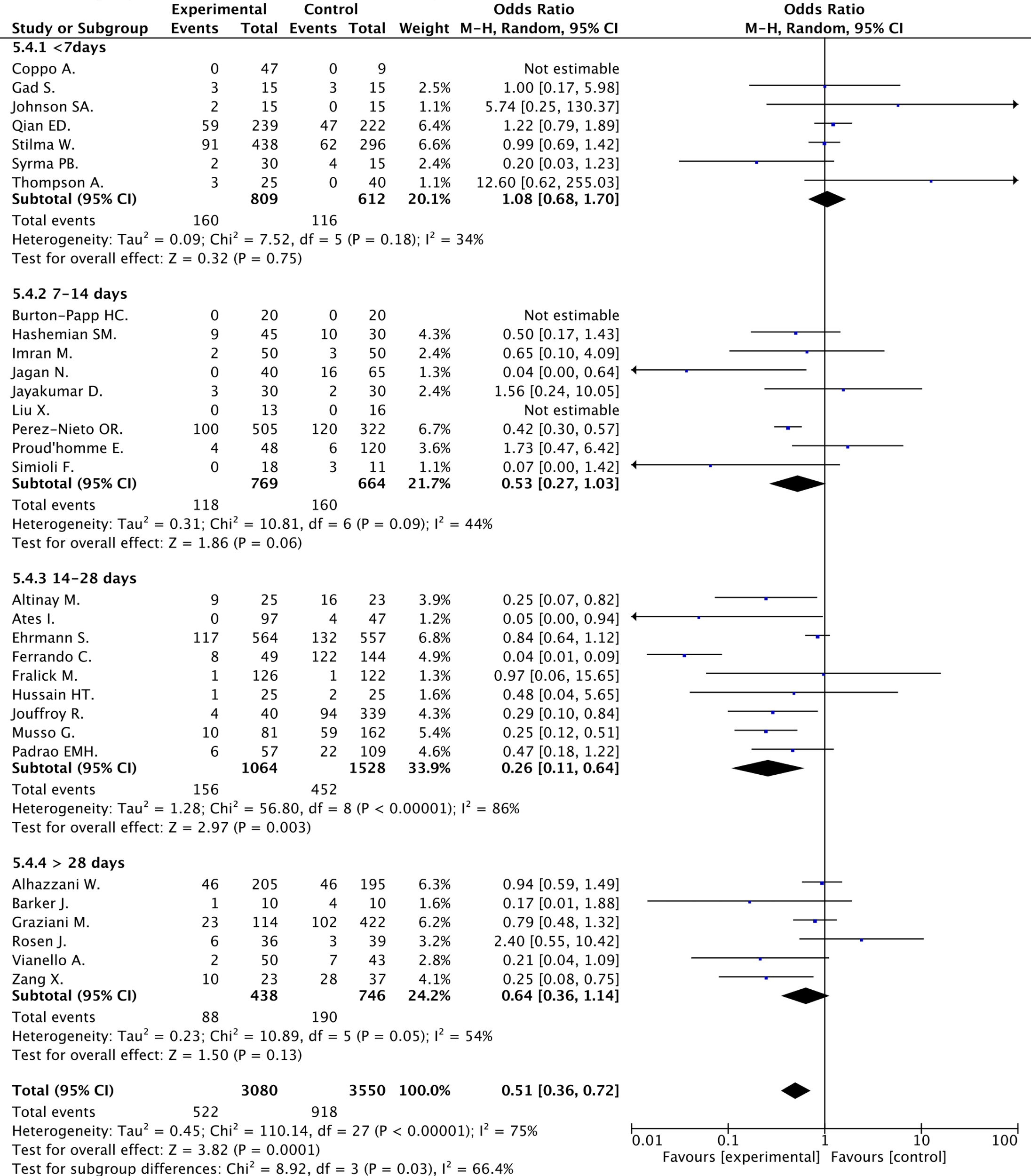

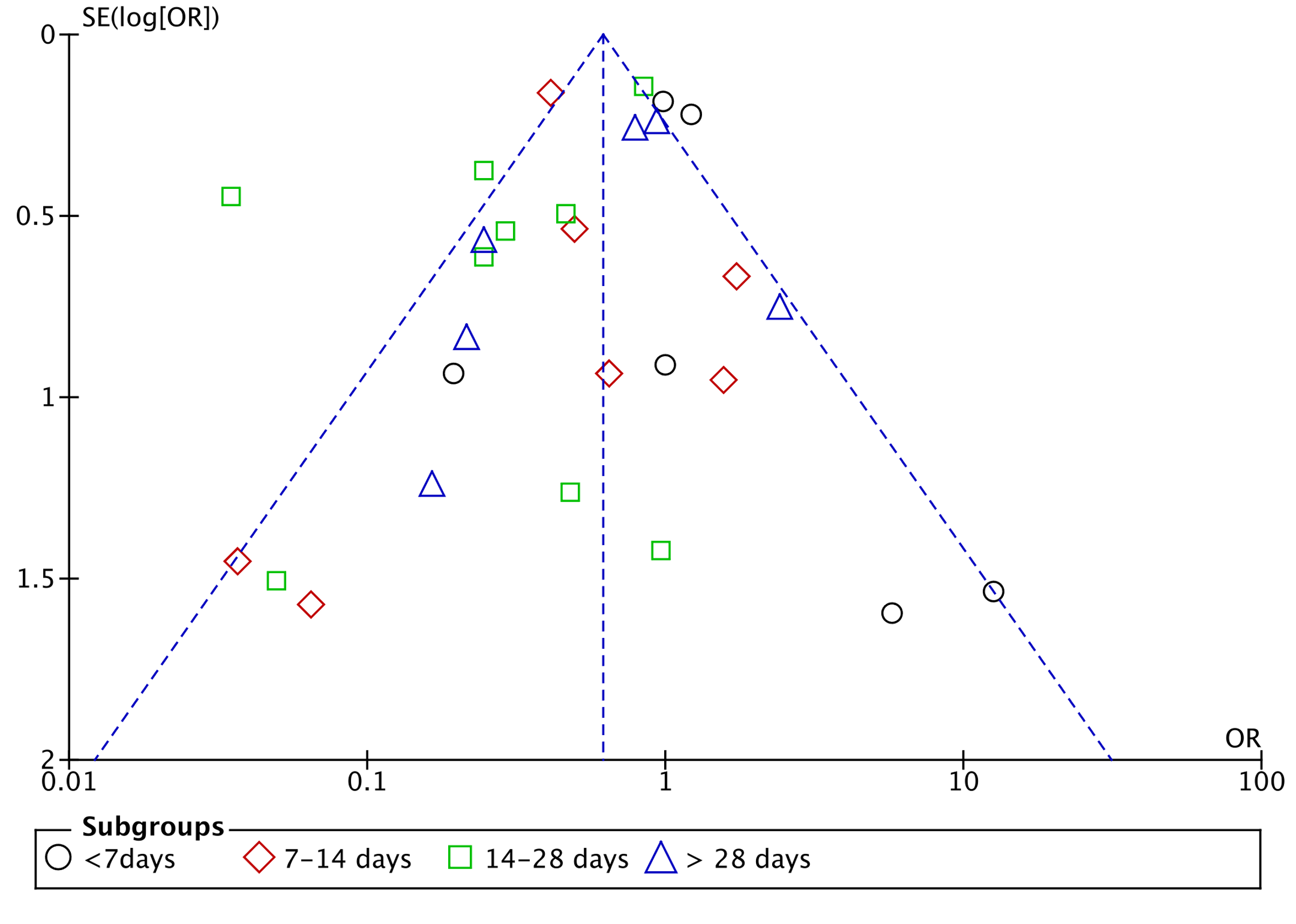


e-figure 12. Forest plot and funnel orotracheal intubation by duration of follow-up


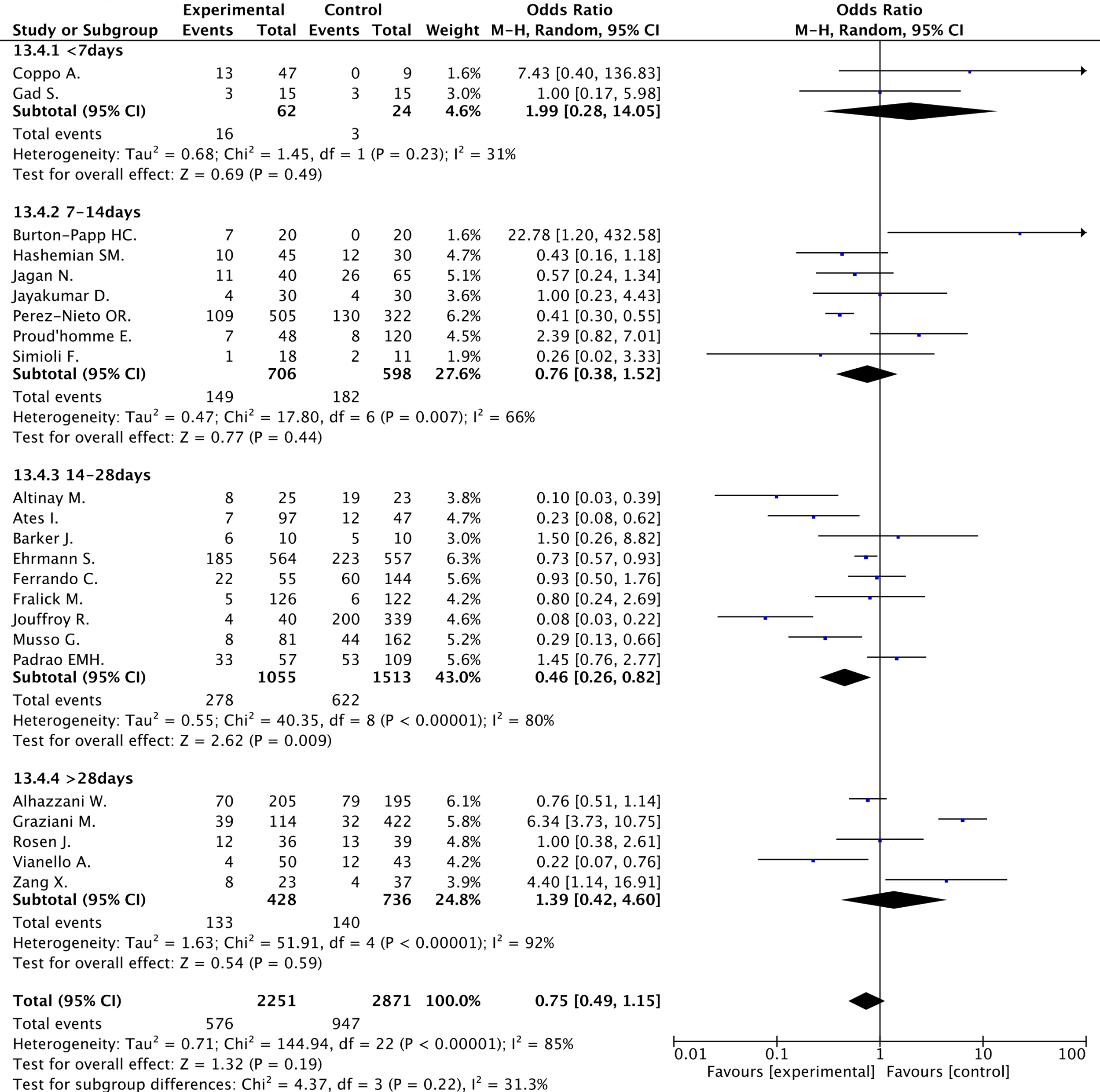

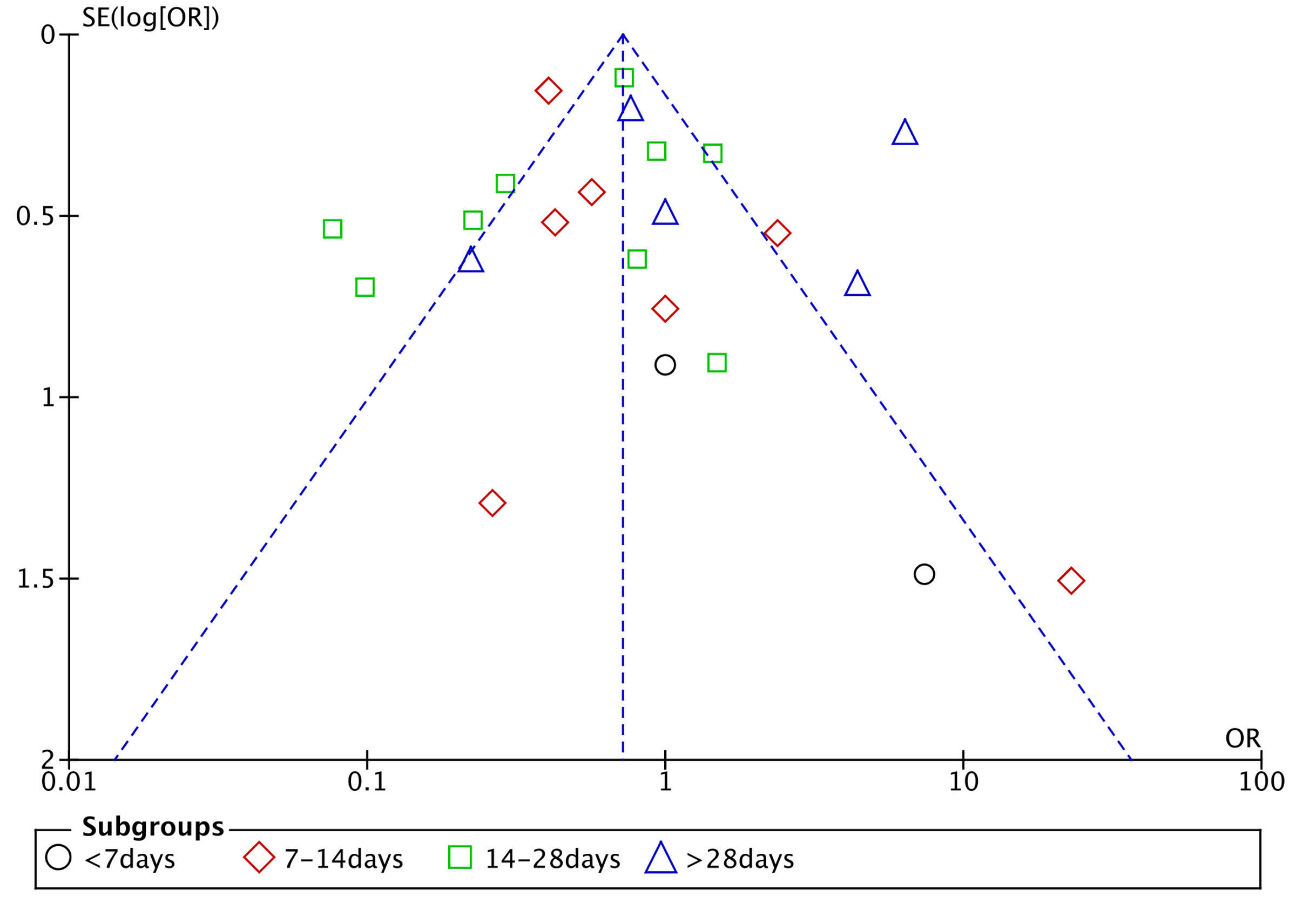


e-figure 13. Forest plot and funnel plot in-hospital death by period of study recruitment


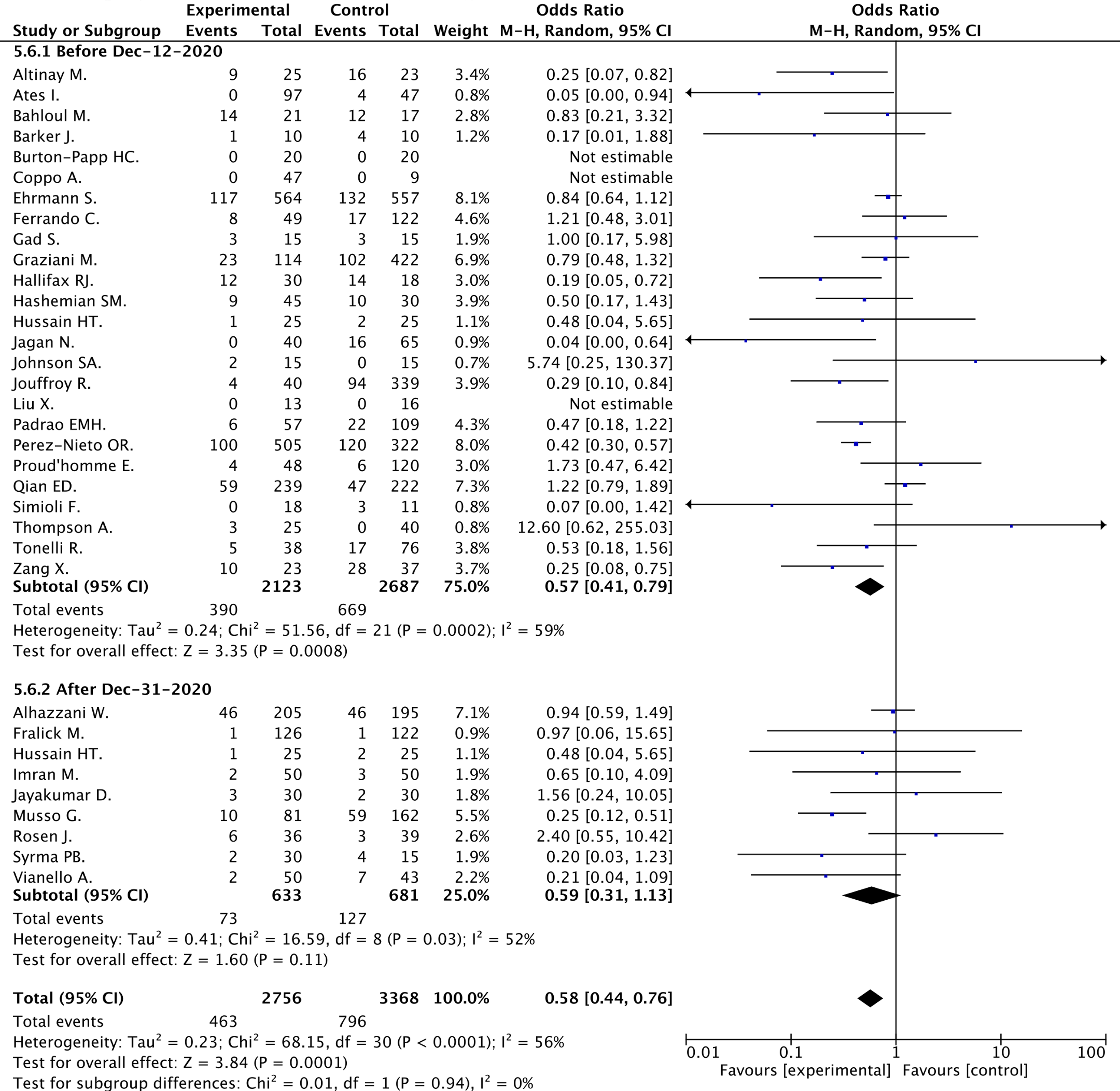

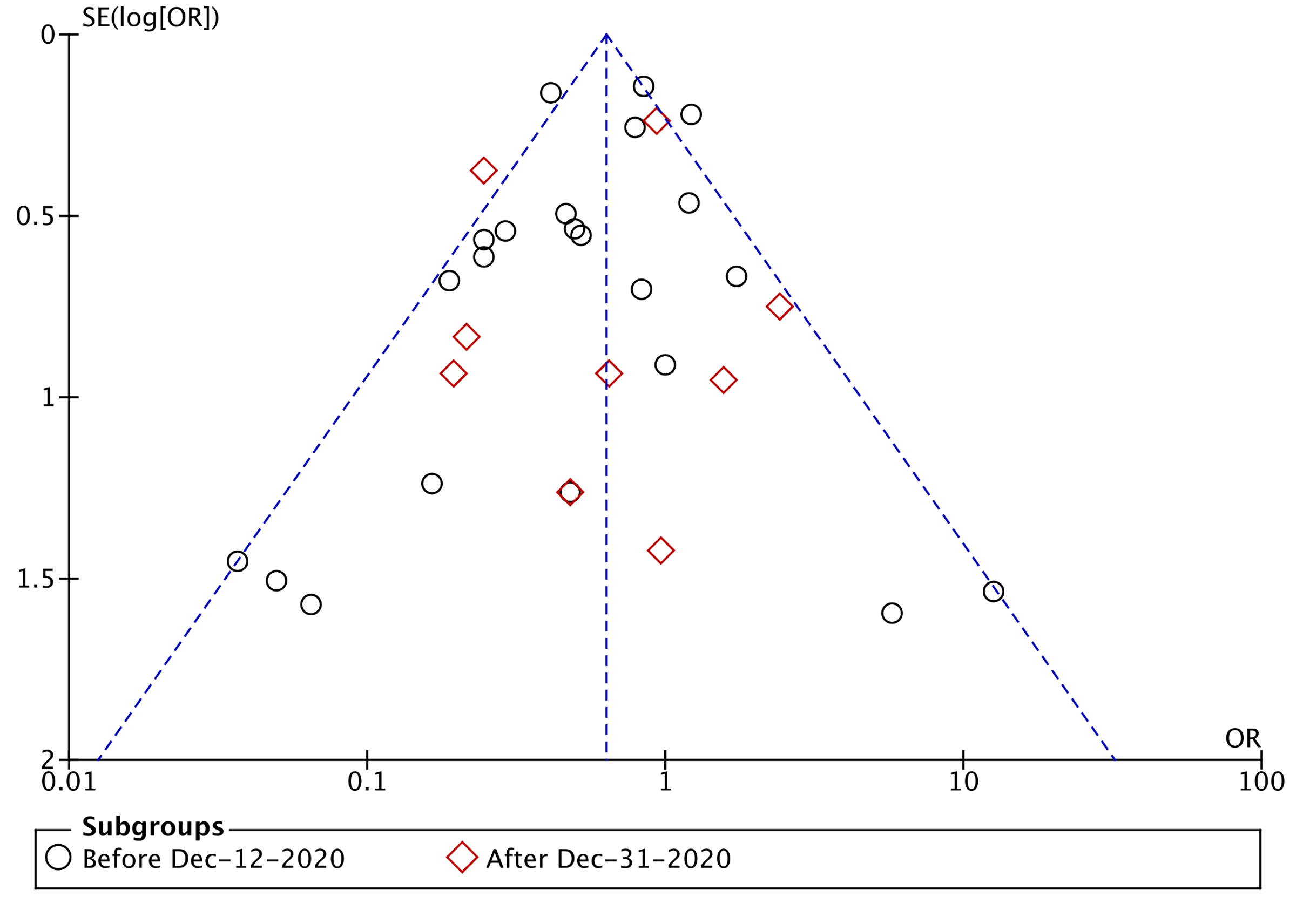


e-figure 14. Forest plot and funnel plot orotracheal intubation by period of study recruitment


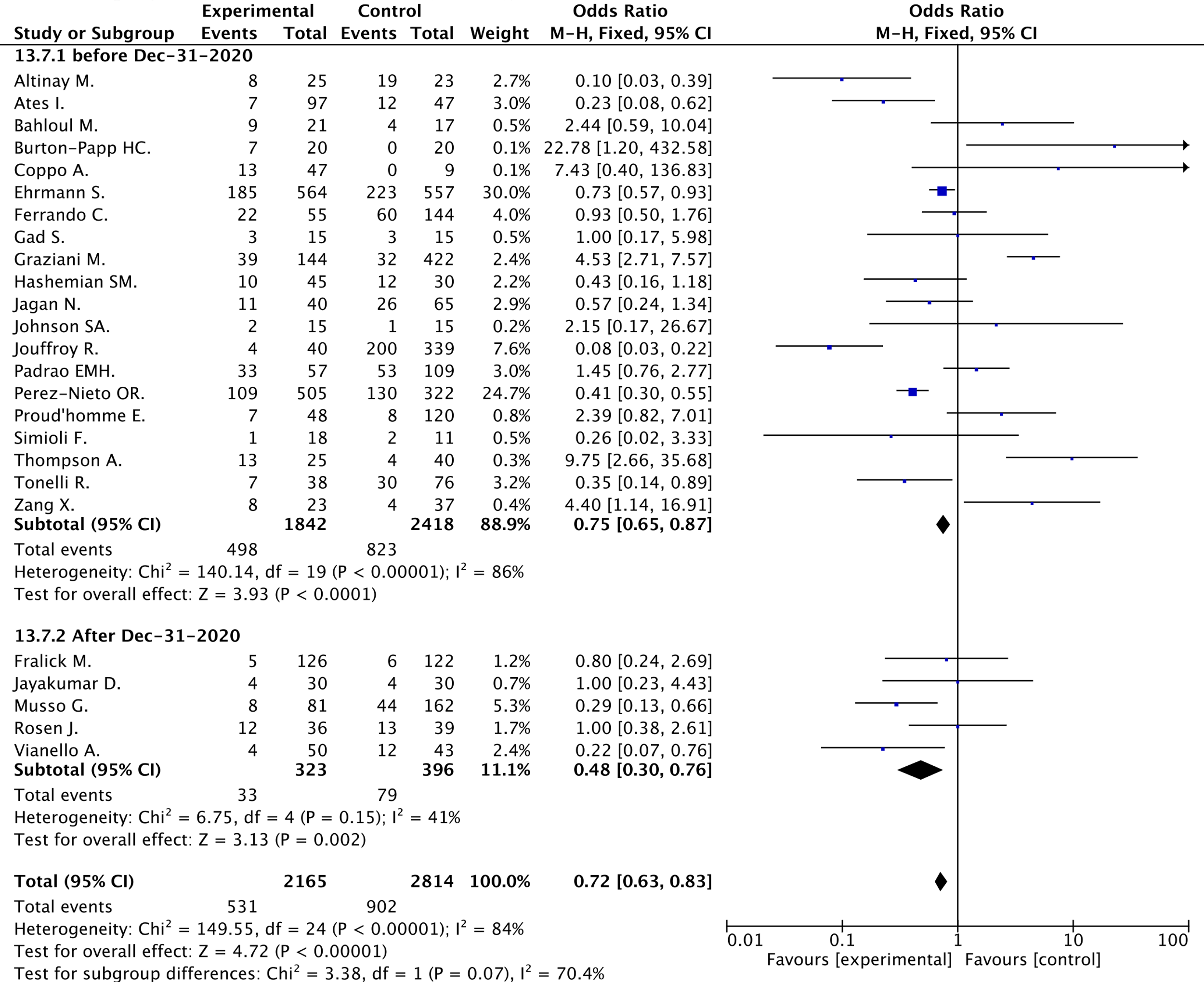

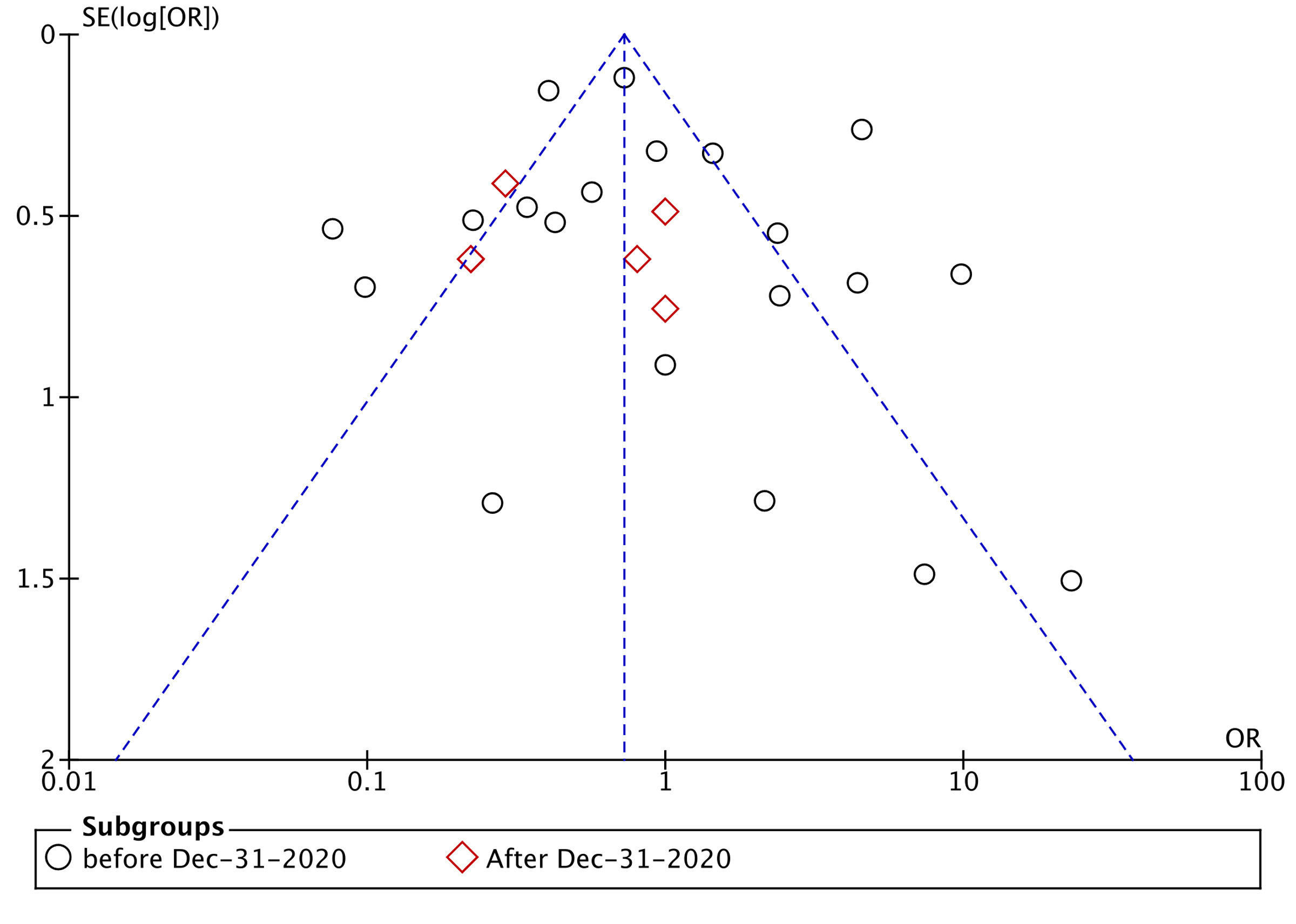


e-figure 15. Forest plot and funnel plot in-hospital death by quality of the studies


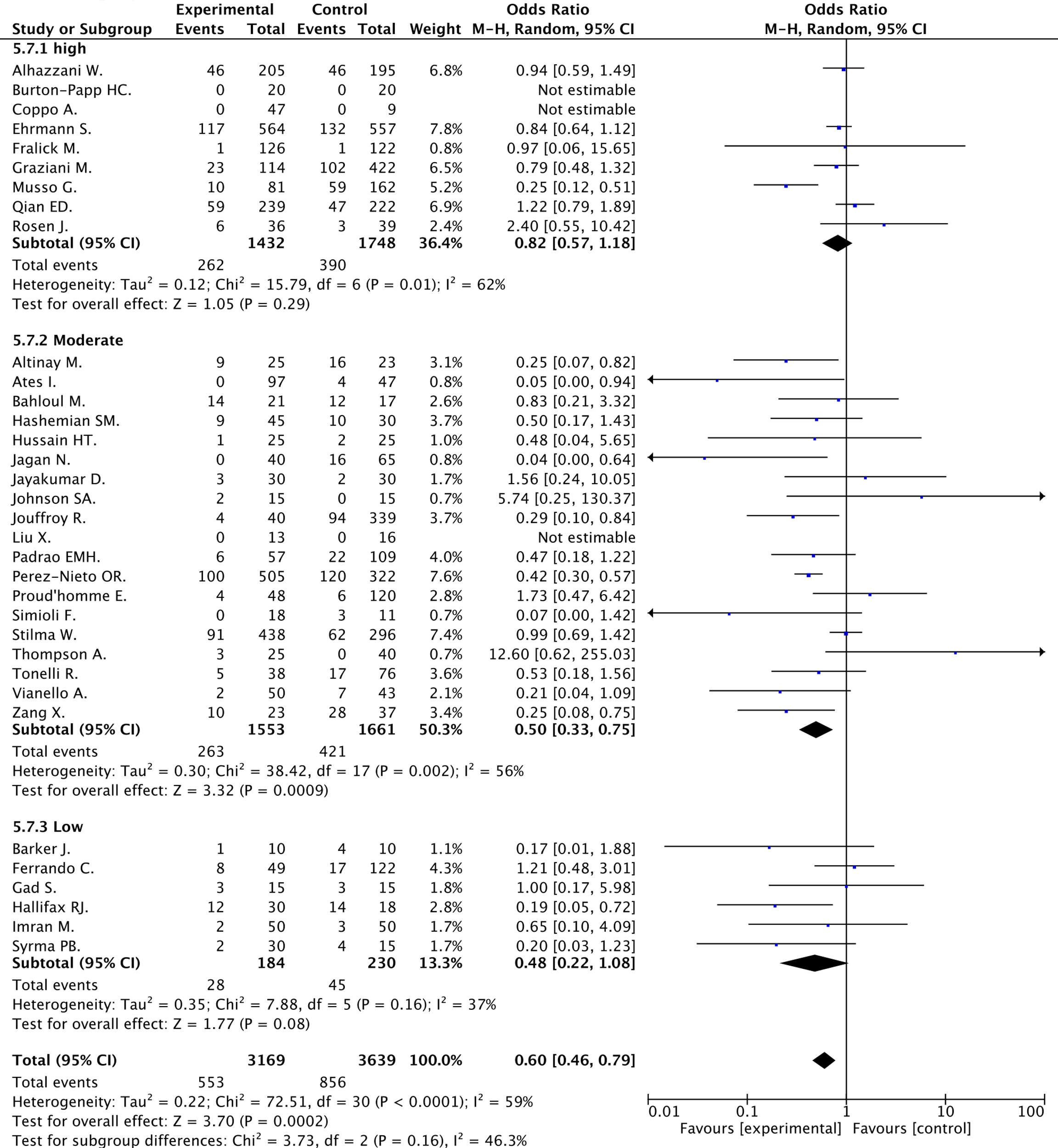

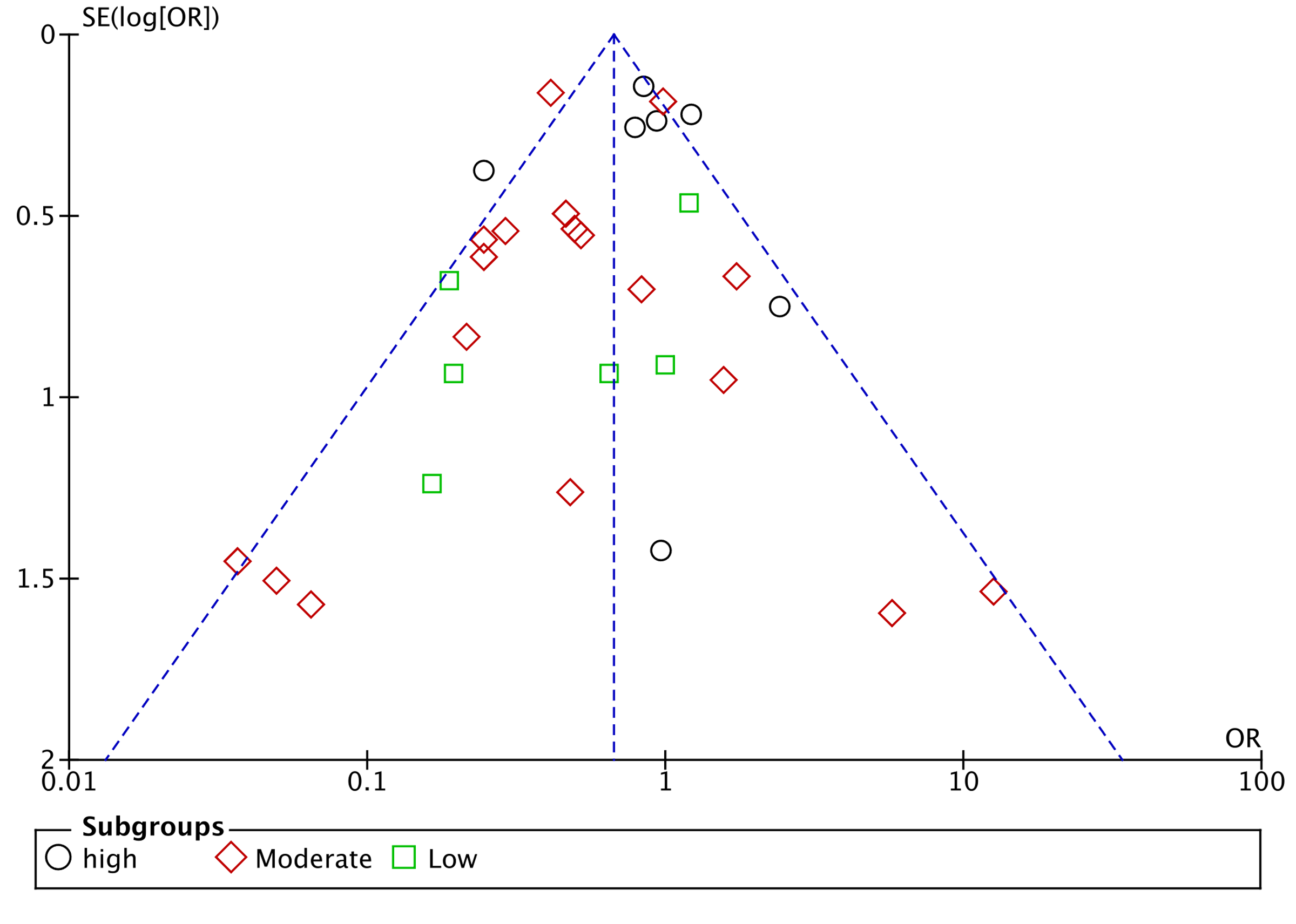


e-figure 16. Forest plot and funnel orotracheal intubation by quality of the studies


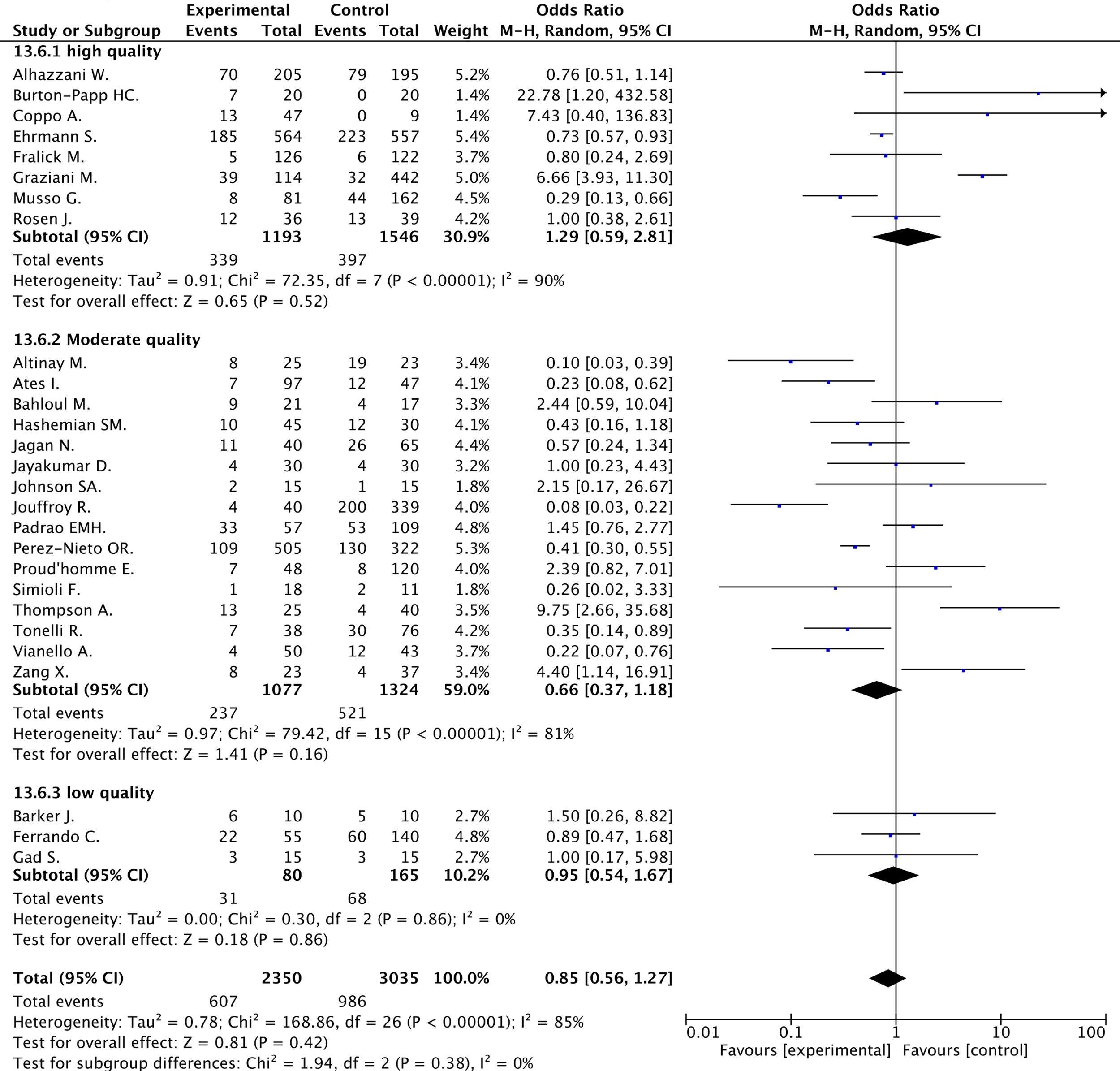

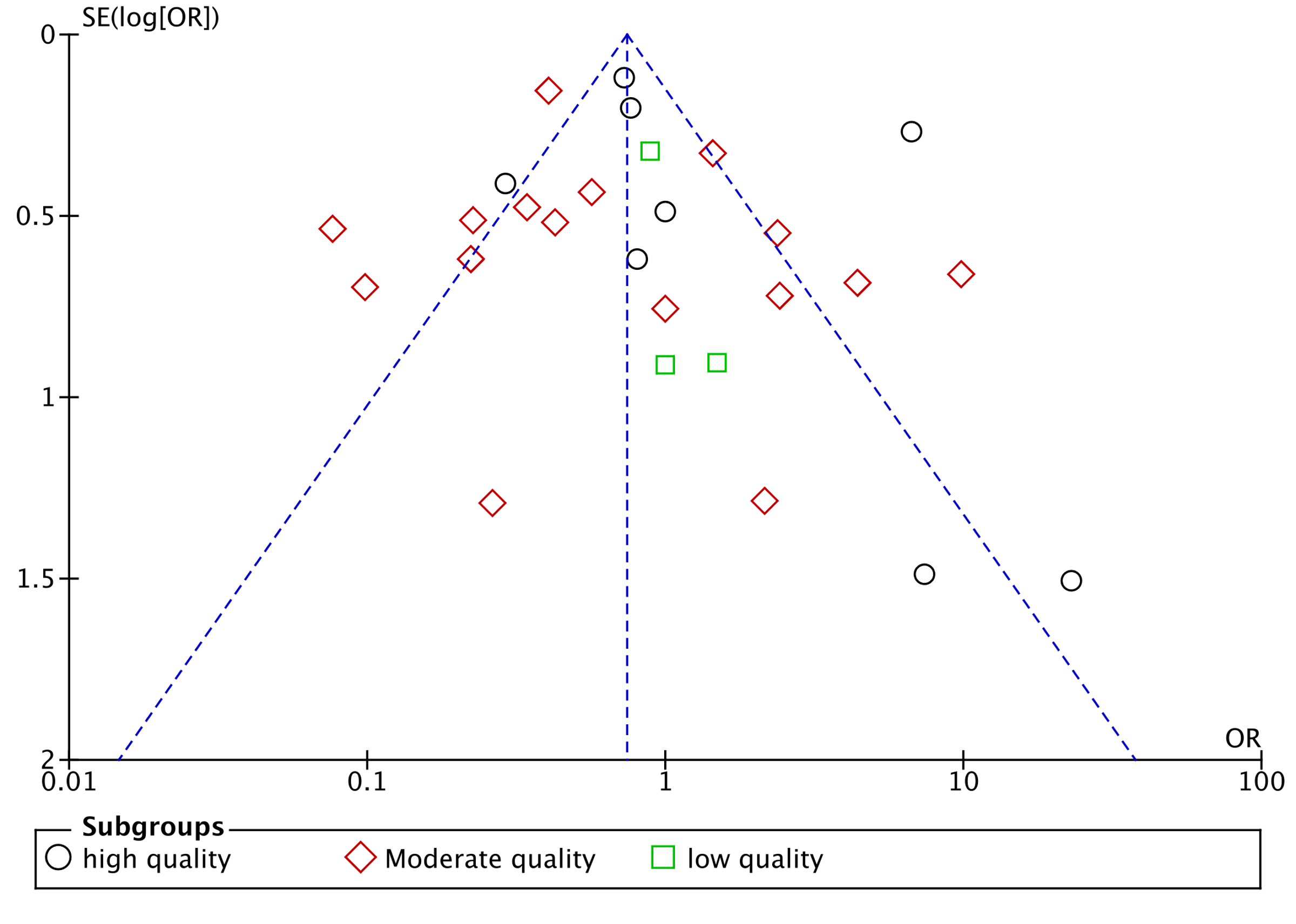

Supplement: Supplementary file 1 — Supplementary file1 (DOCX 27445 KB) [file 11739_2023_3434_MOESM1_ESM.docx]
